# Immune cell type and DNA methylation vary with reproductive status in women: possible pathways for costs of reproduction

**DOI:** 10.1093/emph/eoac003

**Published:** 2022-02-02

**Authors:** Calen P Ryan, Meaghan J Jones, Rachel D Edgar, Nanette R Lee, Michael S Kobor, Thomas W McDade, Christopher W Kuzawa

**Affiliations:** 1 Department of Anthropology, Northwestern University, Evanston, IL 60208, USA; 2 Department of Biochemistry and Medical Genetics, University of Manitoba, Winnipeg, MB R3E 0J9, Canada; 3 Children’s Hospital Research Institute, University of Manitoba, Winnipeg, MB R3E 0J9, Canada; 4 EMBL-EBI, Wellcome Genome Campus, Hinxton CB10 1SD, UK; 5 University of San Carlos Office of Population Studies Foundation Inc., Cebu City 6000, Philippines; 6 BC Children’s Hospital Research Institute, University of British Columbia, Vancouver, BC V5Z 4H4, Canada; 7 Child and Brain Development Program, Canadian Institute for Advanced Research, Toronto, ON M5G 1Z8, Canada; 8 Institute for Policy Research, Northwestern University, Evanston, IL 60208, USA

**Keywords:** pregnancy, breastfeeding, epigenome, immunomethylomics, evolution

## Abstract

**Background:**

Consistent with evolutionarily theorized costs of reproduction (CoR), reproductive history in women is associated with life expectancy and susceptibility to certain cancers, autoimmune disorders and metabolic disease. Immunological changes originating during reproduction may help explain some of these relationships.

**Methodology:**

To explore the potential role of the immune system in female CoR, we characterized leukocyte composition and regulatory processes using DNA methylation (DNAm) in a cross-sectional cohort of young (20–22 years old) women differing in reproductive status.

**Results:**

Compared to nulliparity, pregnancy was characterized by differential methylation at 828 sites, 96% of which were hypomethylated and enriched for genes associated with T-cell activation, innate immunity, pre-eclampsia and neoplasia. Breastfeeding was associated with differential methylation at 1107 sites (71% hypermethylated), enriched for genes involved in metabolism, immune self-recognition and neurogenesis. There were no significant differences in DNAm between nulliparous and parous women. However, compared to nullipara, pregnant women had lower proportions of B, CD4T, CD8T and natural killer (NK) cells, and higher proportions of granulocytes and monocytes. Monocyte counts were lower and NK counts higher among breastfeeding women, and remained so among parous women.

**Implications:**

Our findings point to widespread differences in DNAm during pregnancy and lactation. These effects appear largely transient, but may accumulate with gravidity become detectable as women age. Nulliparous and parous women differed in leukocyte composition, consistent with more persistent effects of reproduction on cell type. These findings support transient (leukocyte DNAm) and persistent (cell composition) changes associated with reproduction in women, illuminating potential pathways contributing to CoR.

**Lay Summary:** Evolutionary theory and epidemiology support costs of reproduction (CoR) to women’s health that may involve changes in immune function. We report differences in immune cell composition and gene regulation during pregnancy and breastfeeding. While many of these differences appear transient, immune cell composition may remain, suggesting mechanisms for female CoR.

## INTRODUCTION

Evolutionary theory posits that resources devoted to reproduction will come at the expense of other functions, including somatic maintenance, accelerating senescence and age-related degenerative decline [1]. Such tradeoffs are expected to take the form of competing molecular or physiological functions that favor fertility and rearing over survival. Evidence for such ‘costs of reproduction’ (CoR) in humans come from both historical and contemporary epidemiological data [2–4], and are strongest among women, for whom the energetic and physiological demands related to reproduction are particularly high [5].

Relative to nulliparous women, mortality from conditions like diabetes, cancer of the uterine cervix, gallbladder disease, kidney disease and hypertension are higher among parous women [3]. Furthermore, grand multiparity (≥5 births) has been linked with increased cardiovascular disease-related mortality [6, 7], kidney cancer [8], late-life cognitive decline [9] and all-cause mortality [4, 10, 11]. In contrast, breastfeeding appears to be protective against obesity, type II diabetes, cardiovascular disease and breast and ovarian cancer [12, 13].

Tradeoffs between reproduction and women’s health may be rooted in many of the core physiological and molecular adaptations to pregnancy and breastfeeding [14]. Characterizing the nature and timing of these adaptations could therefore uncover functional constraints between competing molecular and physiological processes, providing insights into the pathways that link reproductive history to women’s health. Hemochorial placentation in humans puts the fetal chorion in direct contact with the maternal blood supply. This allows unimpeded transfer of glucose and other resources to the developing fetus, but can also give rise to fetal manipulation of endocrine signaling, metabolic regulation and hemodynamic control that can have long-term deleterious impacts on maternal health [15]. Highly invasive placentation also presents an immunological conundrum—during pregnancy, the maternal body must shift from acquired to innate immunity to retain immunocompetence against pathogens and infection while accommodating a semi-allogenic conceptus [16]. Pregnancy is also accompanied by involution of the thymus, an organ responsible for the maturation of T-cells that are central to adaptive immunity, while implantation and labor are both pro-inflammatory events [17]. The shift from acquired to innate immunity that accompanies pregnancy increases inflammation, oxidative stress and cellular damage [14, 16]. During breastfeeding, a high maternal metabolic burden is necessary to nourish the comparatively under-developed and energetically costly infant brain [18]. The maternal immune system during breastfeeding is also highly active, clearing the body of fetal cells and DNA absorbed during pregnancy (i.e. fetal microchimerism), while producing a select set of immunogenic compounds that are vital to infant development [19]. These immunological changes—along with the metabolic, circulatory and endocrine adaptations necessary for reproduction—may help explain why the risk for cardiovascular disease [20], kidney disease [21], cognitive decline [22] and cancer [23] are elevated with increasing parity, and may be an important bridge between reproduction and women’s long-term health [14, 24, 25].

A role for the maternal immune system in long-term health costs related to reproduction could include (i) changes to immune cell composition in the maternal circulation during pregnancy and/or breastfeeding, (ii) changes in gene regulation in the immune cells themselves during these reproductive stages or (iii) both. Flow cytometry has demonstrated differences in the proportions of memory T-cells [26], monocytes [27], granulocytes and natural killer (NK) cells [28] during pregnancy, supporting broad immunological shifts with reproduction. Other work has documented differences in inflammatory biomarkers among pregnant women [29], consistent with regulatory changes in cytokine production that accompany a shift from adaptive to innate immunity. Some of these immunological effects may be cumulative and persistent [24, 30, 31], but there remain considerable gaps in our understanding of how alterations in immune profiles and regulatory control might be tied to CoR in women.

As with much research on the biology of reproduction in women, the study of maternal immunity and reproduction is often framed in relation to effects on infant health and risk for pregnancy complications. As a result, research on immunity during pregnancy has tended to focus on the fetomaternal interface [32], with changes in gene regulation within immune cells receiving less attention [33–35]. Breastfeeding may also be linked to autoinflammatory processes and disease [36], yet studies examining the potential long-term effects of breastfeeding on maternal immunological disorders are rare. Work in this area has also been conducted almost exclusively in relatively affluent western nations—where reproductive effort may be low and exposure to environmental pathogens and microbes limited—despite evidence that these socioecological contexts can affect the maternal immune response to reproduction [37].

To clarify the immunological processes associated with reproduction, we used the Illumina BeadChip 450k Array to examine genome-wide blood leukocyte DNA methylation (DNAm) in 394 women who were similar in age (20–21 years), but varied in reproductive status (nulliparous, pregnant, breastfeeding and parous) at the time of measurement. The women in this study are participants in the Cebu Longitudinal Health and Nutrition Survey, a long-term study of health and life histories in the Metropolitan Cebu Area, Philippines [38, 39]. DNAm is a biochemical process that reflects chromatin accessibility and transcriptional activity, providing a tool for studying gene–environment interactions that often underlie development, aging and disease [40]. When applied to blood, DNAm can also be used to bioinformatically impute proportions of circulating leukocytes [41]. We capitalized on these attributes of DNAm to explore systemic differences in immune function by looking at cell composition, as well as more targeted changes in regulatory activity within the immune cells themselves. Previous work in this [24] and other [42] populations has documented accelerated DNAm and telomere-based measures of cellular aging with gravidity and parity. DNAm-based measures of cellular aging have themselves been associated with mortality and disease risk [43], suggesting that changes in DNAm at certain loci may be a link or causal marker of the fundamental processes connecting reproductive history and women’s long-term health. However, the changes in DNAm that accompany pregnancy and breastfeeding, and how they relate to each other, are still not well-characterized.

We hypothesized that reproduction in this sample would be associated with differences in immune function at both the systemic and molecular level, consistent with a possible role of immune changes to CoR. During pregnancy, we expected shifts in immune cell composition and DNAm to reflect the documented reprioritization of innate over acquired immunity, as well as widespread hypomethylation with pregnancy, consistent with previously reported increases in gene expression throughout gestation [33]. We anticipated differences in DNAm during breastfeeding to be reflective of the higher metabolic demands of lactation, and changes reflective of the positive effect of breastfeeding on breast cancer risk. Finally, given long-term effects of reproductive history on women’s health, we expected a subset of differences in DNAm and cell composition to exist between nulliparous and parous women, consistent with a persistent biological cost of reproduction in women.

## METHODOLOGY

### Participants and study design

Data come from the Cebu Longitudinal Health and Nutrition Survey (CLHNS), a birth cohort study in Metropolitan Cebu, Philippines that began with enrollment of 3327 pregnant mothers in 1983–84. This study focuses on the offspring, who were 20–22 years of age in 2005 when blood for DNAm was collected. Rates of refusal during initial recruitment were low (<4%), and attrition in the CLHNS is due primarily to factors related to out-migration [38]. Written informed consent was obtained from all participants with oversight by the Institutional Review Boards of the University of North Carolina at Chapel Hill and Northwestern University.

A total of 392 women were included in this study. These women were drawn from a subsample of 1759 women who provided a blood sample in 2005 and later participated in a pregnancy tracking study. Reproductive histories were based on an in-home survey administered by a trained interviewer in 2007. The survey included questions about each known pregnancy, its duration, prenatal care, birth outcome (e.g. live birth, miscarriage, stillbirth and twins) and breastfeeding initiation and termination. Date of conception was inferred based on pregnancy duration and date of pregnancy termination (i.e. birth, miscarriage, etc.). When participants could not recall the day of pregnancy termination, the 15th of the month was used. Based on these records, women were classified as pregnant, breastfeeding, parous (but not breastfeeding or pregnant) and nulliparous. Women were classified as ‘pregnant’ when the blood sample date fell between the date of conception and the date of pregnancy termination; as ‘breastfeeding’ when blood sample date fell between the initiation of breastfeeding and the termination of breastfeeding. Women with pregnancies prior to the date of blood sample, but who were not otherwise breastfeeding or pregnant were classified as ‘parous’. Women who reported never having been pregnant for any duration up to and during the time of the blood sample were classified as ‘nulliparous’. Two women who were simultaneously pregnant and breastfeeding were classified as ‘pregnant’.

### DNAm and statistical analysis

Blood collection, DNA extraction and DNAm analysis were conducted using methods described previously [24] and in more detail in the supplementary material. Briefly, DNAm was measured on the Illumina HumanMethylation450 Bead Chip (Illumina Inc., San Diego, CA), and run through standard pre-processing and quality control procedures in Genome Studio and R. Immune cell composition was imputed using DNAm based on Reference [41], and DNAm associated with immune cell variance was removed for genome-wide analyses using a linear regression approach. A total of 434 728 probes passed quality control procedures. Invariable sites were filtered out to maximize statistical power, leaving a subset of 110 631 probes for analysis. Models were fit using linear regression, and false discovery rate was controlled for using the method of Benjamini and Hochberg. The following contrasts were made: nulliparous-pregnant, nulliparous-breastfeeding, parous-pregnant, parous-breastfeeding and nulliparous-parous. To control for unmeasured environmental and genetic factors, all models included smoking status, two principal components of genetic variation (genetic PC-scores) based on multidimensional scaling using Euclidean distance and a composite measure of socioeconomic status (SES) (see the supplementary material for more references and details on the derivation of these measures). We further examined differences between reproductive status groups using a ‘bumphunting’ approach to detect differentially methylated regions [44]. The parameters used and results of these methods are described in detail in the supplementary material.

### Gene ranking and functional annotation

Each probe was annotated using UCSC_RefGene_Name column from the Illumina annotation file. Genes were then ranked using average standardized −log10 *P*-value and log10 absolute delta-beta values for each gene. The resulting rank was used for Table 2 and the functional enrichment analysis using gene ontology (GO). Gene functions were determined using openly accessible compendia and curated databases. A total of 17 303 annotated genes associated with the variable probes were used as the enrichment background list. Enrichment of GO terms in the ranked list of differentially methylated genes was tested using the receiver operator characteristic (ROC) method in ErmineJ [45]. Because ROC is based on the relative ranking of genes, significant enrichment for biological pathways is possible even when there are no differentially methylated sites within a given gene. Networks were constructed using EnrichmentMap in Cytoscape based on ErmineJ output. Additional references and details on the parameters used for enrichment and network construction are provided in the supplementary material.

## RESULTS

### Descriptive statistics

Women of different reproductive statuses did not differ in age or genetic PC-scores 1 and 2 (*P* = 0.43 and 0.68, respectively), but did differ by smoking status (*P* = 0.007) and SES (Table 1). While no pregnant or breastfeeding women smoked, three nulliparous women and seven parous women reported smoking. SES was higher among nulliparous women compared to other reproductive categories (*F*_3,388_ = 3.63, *P* = 0.0132). Hierarchical clustering by Euclidian distance did not reveal any grouping by SES quartiles, smoking or genetic PC-score quartiles, suggesting that these covariates were not confounding in our analysis of reproductive status.

**Table 1.** Descriptive statistics of 392 young (20–22 years old) women of varying reproductive statuses participating in the Cebu Longitudinal Health and Nutrition Survey (CLHNS)

	Nulliparous (*N* = 176)	Pregnant (*N* = 69)	Breastfeeding (*N* = 60)	Parous (*N* = 87)	Total (*N* = 392)	*P*-value
Age (years)						
Mean (SD)	21.65 (0.35)	21.66 (0.33)	21.72 (0.32)	21.67 (0.36)	21.67 (0.35)	0.636^a^
Range	20.84–22.44	20.90–22.42	21.05–22.47	20.88–22.40	20.84–22.47	
Gravidity						
Mean (SD)	0 (0)	1.83 (1.07)	1.73 (0.76)	1.41 (0.69)	0.9 (1.04)	<0.001^a^
Range	0–0	1–5	1–4	1–4	0–5	
SES-score						
Mean (SD)	0.05 (1.35)	−0.50 (1.31)	−0.38 (1.50)	−0.34 (1.41)	−0.20 (1.40)	0.013^a^
Range	−2.75 to 3.95	−2.75 to 3.19	−3.27 to 3.81	−3.10 to 3.90	−3.27 to 3.95	
Genetic PC-score 1						
Mean (SD)	0.16 (9.01)	−0.42 (8.82)	1.93 (7.55)	0.10 (7.75)	0.32 (8.50)	0.423^a^
Range	−19.94 to 24.04	−21.15 to 15.84	−12.54 to 22.85	−24.95 to 18.70	−24.95 to 24.04	
Genetic PC-score 2						
Mean (SD)	−0.20 (8.06)	0.46 (7.93)	−0.30 (7.47)	0.91 (6.94)	0.15 (7.70)	0.681^a^
Range	−20.48 to 20.33	−17.07 to 17.50	−15.41 to 17.56	−17.92 to 19.17	−20.48 to 20.33	
Smoking status (1 = ‘yes’)						
Number (%)	3 (2%)	0	0	7 (8%)	10 (3%)	0.007^b^

More details on the derivation of the socioeconomic status composite score (SES-score) and the genetic principal components (genetic PC-scores 1 and 2) can be found in the supplementary material.

a Linear model ANOVA.

b Fisher’s exact test for count data.

### Immune cell composition by reproductive status

**Figure 1.**
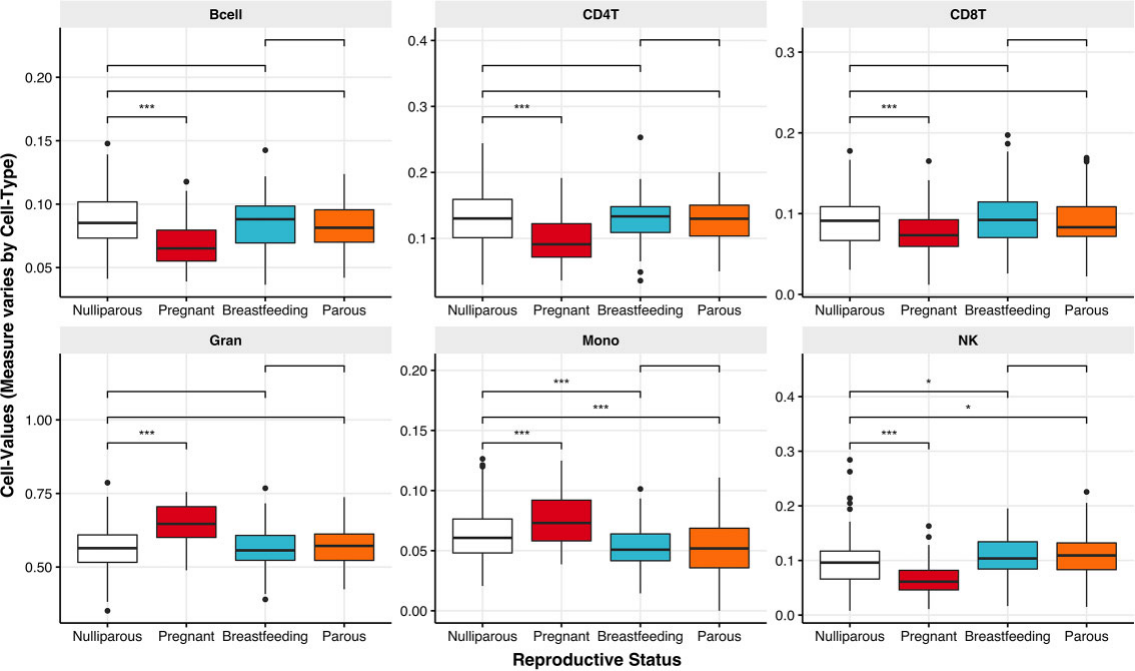
Imputed immune cell composition among women of differing reproductive status B lymphocytes (B-cells), CDT4 lymphocytes (CD4T), CD8T lymphocytes (CD8T), granulocytes (Gran), monocytes (Mono) and NK cells. Note: *P < 0.05; **P < 0.01; ***P < 0.001.

We used reference-based deconvolution methods to infer cell-type proportions from DNAm [41], allowing us to quantify differences in the composition of circulating leukocytes with reproductive status. Controlling for smoking status, SES, PC-scores of genetic variation and age at blood draw, reproductive status predicted cell proportions across all measured cell types (Fig. 1 and Supplementary Table S1). Compared to nulliparous women, the proportions of B-cells, CD4T, CD8T and NK cells were lower during pregnancy, while the proportions of granulocytes and monocytes were higher (Fig. 1 and Supplementary Table S1). Similar differences were observed when parous women were used as the reference group (Supplementary Table S2). Most of the differences in cell composition associated with pregnancy appear to be resolved during breastfeeding: the proportion of B-cells, CD4T, CD8T and granulocytes were similar among nulliparous and parous women (Fig. 1 and Supplementary Tables S1 and S2). However, the proportions of monocytes and NK cells were lower and higher, respectively, among breastfeeding women compared to nulliparous women, and remained so for parous women, suggesting potentially persistent changes in these cell types that accompany reproduction (Fig. 1). To test whether these differences were persistent, we examined whether cell composition varied in relation to time since parturition among parous women. We found no evidence that cell composition differed among women varying in time since parturition, up to 5.5 years after the end of pregnancy, supporting the interpretation that differences in monocytes and NK cell counts among parous women were persistent (Supplementary Fig. S1 and Table S3). Including a polynomial term for non-linear changes in cell type with time since parturition did not change these findings (Supplementary Table S4).

### Genome-wide DNAm by reproductive status

**Figure 2.**
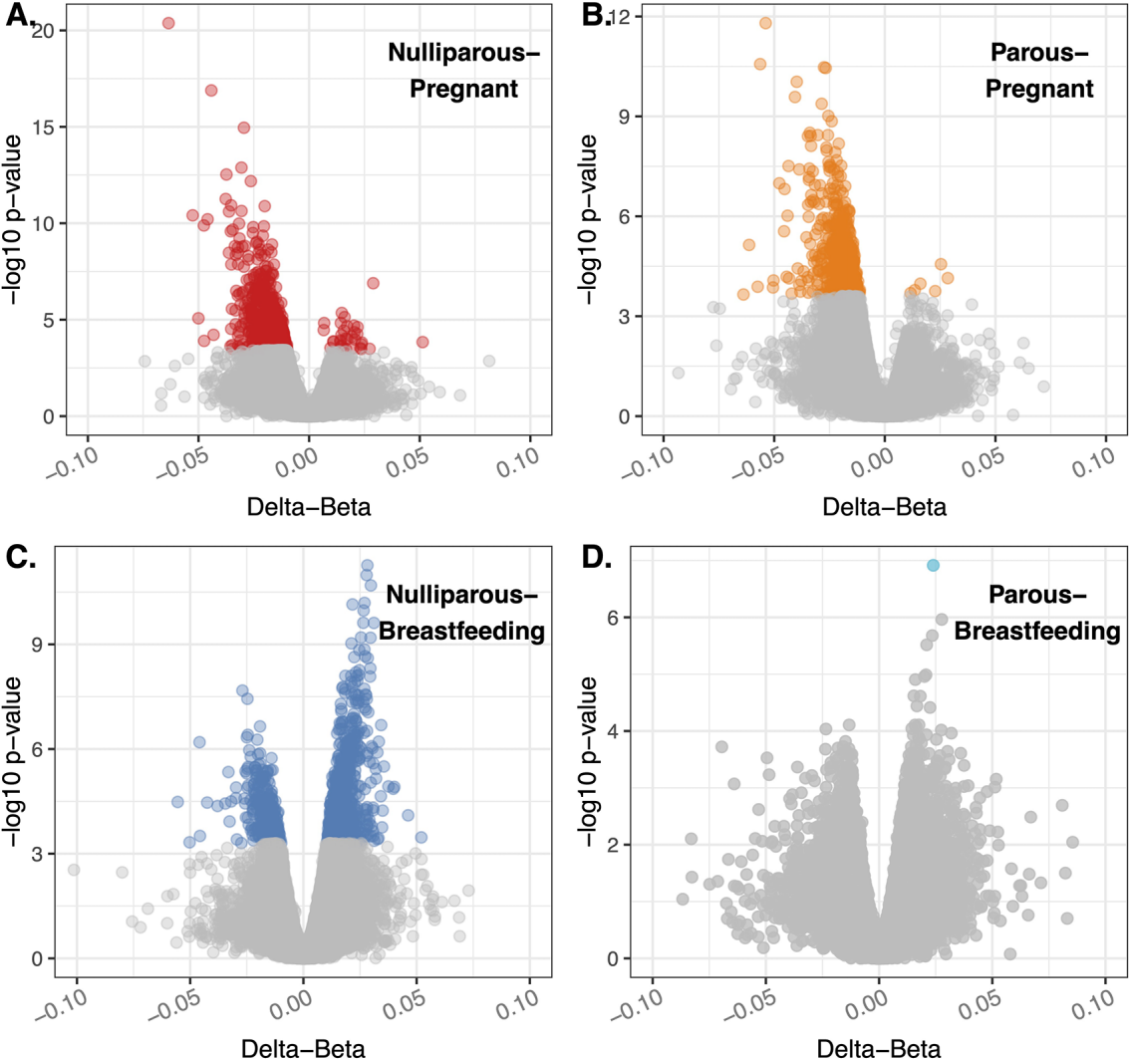
Volcano plots of differences in DNAm (delta-beta) by negative log10 *P*-value between women of differing reproductive status Comparisons between nulliparous-pregnant (**A**), parous-pregnant (**B**), nulliparous-breastfeeding (**C**) and parous-breastfeeding (**D**) are shown, where nulliparous and parous form the reference groups. Sites that are differentially methylated after false discovery correction are colored. *Y*-axis scale varies by plot. The comparison between nulliparous and parous women did not differ significantly for any individual CpG sites after FDR correction, and is not shown.

**Figure 3.**
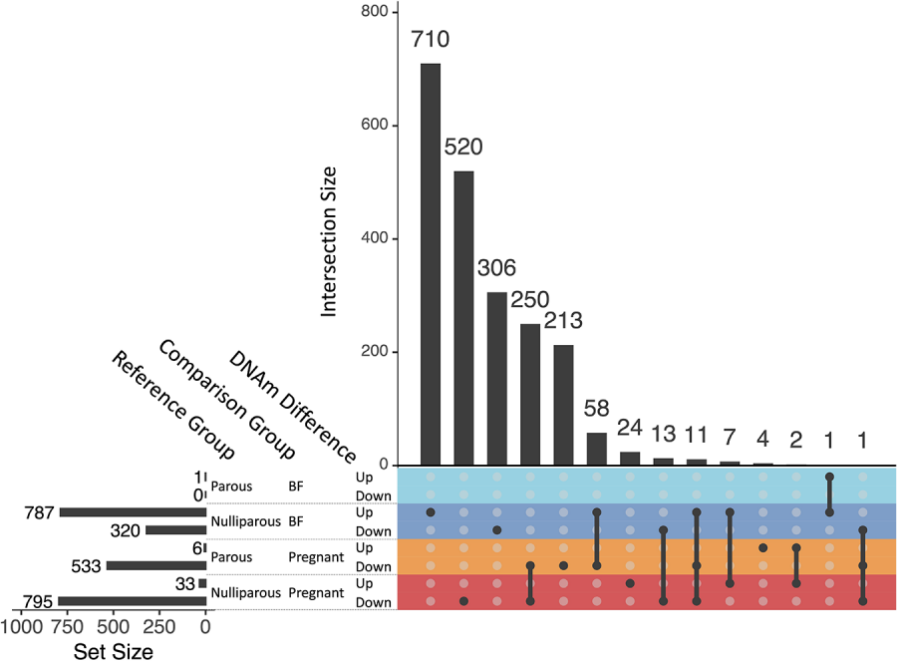
Upset plot showing overlap of DMPs between women of differing reproductive status Single points represent distinct DMPs, whereas connected points represent overlapping DMPs between groups. Differences are categorized as up or down methylated relative to the reference group (nulliparous or parous women), with numbers of DMPs for each category are displayed on the top of the graph.

To further explore the potential regulatory changes within immune cells themselves during pregnancy and breastfeeding, we looked at differences in DNAm at 110 631 CpG sites across the genome, correcting for blood cell composition. Compared to nulliparous women, differential methylation among pregnant women was observed in a total of 828 CpG loci spanning 533 annotated genes (CpG/gene—range: 1–19, median =3). Of these 828 differentially methylated positions (DMPs), 96% (795/828) had lower levels of methylation during pregnancy (Figs 2A and 3 and Supplementary Table S5). Compared to parous women, 539 CpG loci were differentially methylated among pregnant women, spanning 352 annotated genes (CpG/gene—range: 1–25, median =1). Of these 539 DMPs, 99% (533/539) had lower levels of methylation during pregnancy (Figs 2B and 3 and Supplementary Table S5). Roughly half (49%, 264/539) of the DMPs when comparing pregnant and parous women overlapped with the comparison between pregnant and nulliparous women (Fig. 3). All of these were concordant for direction of methylation differences found for pregnancy (Fig. 3).

Relative to nulliparous women, differential methylation among currently breastfeeding women was observed in a total of 1107 CpG loci in 849 annotated genes (CpG/gene—range: 1–6, median =1). Only 8% (90/1107) of DMPs found among breastfeeding women overlapped with DMPs noted above as associated with pregnancy, with 77% (69/90) of these discordant in direction of methylation compared to nulliparity (Fig. 3). In contrast with pregnancy, breastfeeding was associated with greater methylation relative to nulliparity, with 71% (787/1107) of DMPs being more methylated among breastfeeding women (Fig. 2C). A comparison between parous and breastfeeding women only revealed one DMP (Fig. 2D). This site (cg07549715) is located in the gonadotropin releasing hormone 2 (GNRH2) gene and was one of the 1107 DMPs found between nulliparity and breastfeeding. We did not detect statistically significant DMPs between nulliparous and parous women after correcting for false discovery rate. Plots and descriptions of the differences in DNAm between reproductive group for the top-4 CpG sites (ranked by absolute delta-β values) for each comparison are available in Supplementary Figs S2–S6.

### Gene ranking, functional enrichment and network analysis

Genes were ranked based on the sum of maximum standardized −log10 *P*-values and absolute delta-β for each gene, such that the highest ranked genes are those for which both the *P*-values were lowest and differences between groups were highest. The top 10 ranked genes for each comparison of reproductive status are provided in Table 2. Nulliparous-pregnant and parous-pregnant overlapped in 7 of their top 10 genes, mirroring the large overlap in DMPs between nulliparous-pregnant and parous-pregnant women (Table 2). Among these were CLEC2D (a gene involved in innate immunity through the NK cell C-type lectin receptor), TNFSF10 (a cytokine tied to apoptosis), CUEDC1 (a widely expressed gene that is also tied to cervical cancer and pre-eclampsia), SBNO2 (a transcriptional co-regulator that counteracts the inflammatory action of IL-10) and ZEB2 (a master regulator of the epithelial–mesenchymal transition, key to embryo implantation and tissue regeneration, fibrosis and neoplasia). A large number of these genes (8/10 and 9/10 comparing pregnancy to nulliparity and parity, respectively) overlapped with genes containing pregnancy-associated DMPs described by Gruzieva *et al.* [34] (Table 2). These patterns were partly reflected in enrichment networks comparing pregnant women with nulliparous and parous women, which showed evidence of differences in T-cell activation and cell-mediated cytotoxicity (Supplementary Figs S7 and S8).

**Table 2.** Top 10 genes differing between reproductive status groups scored using the mean sum of standardized −log10 *P*-values and absolute delta-β (group differences)

Nulliparous-pregnant		Parous-pregnant		Nulliparous-breastfeeding		Parous-breastfeeding		Nulliparous-parous	
Gene	Score	Gene	Score	Gene	Score	Gene	Score	Gene	Score
*CLEC2D*	10.836	*CLEC2D*	6.972	*DNAH10*	5.700	*GNRH2*	5.741	*RTP2*	5.23
*TNFSF10^††^*	8.916	*ZEB2^††^*	6.331	*FAM193B*	5.561	*CPM*	5.044	*NIPBL*	4.98
*CUEDC1^††^*	7.738	*SBNO2* **^*†*^** *^†^*	5.938	*MLNR*	5.455	*FAM13A*	4.725	*FBX011*	4.92
*CCR7*	6.679	*NADK^†^*	5.859	*SLC38A10*	5.162	*ANKFY1*	4.533	*TMED2*	4.48
*SBNO2^††^*	6.514	*RORC^†^*	5.623	*AMBRA1*	5.050	*LCP2*	4.094	*BSPH1*	4.09
*BMP1^†^*	5.859	*NACC2^†^*	5.324	*KIAA0146*	5.024	*DNAH10*	4.053	*CWC15*	4.03
*TMEM49^††^*	5.828	*CUEDC1* **^*†*^** *^†^*	5.068	*ASPRV1*	4.966	*ST5*	3.888	*KDM4D*	4.03
*NADK^†^*	5.717	*GGT6^†^*	4.948	*VTI1A*	4.736	*LSM12*	3.719	*ARMC7*	3.86
*CISH^†^*	5.688	*TNFSF10^††^*	4.946	*IL1R1*	4.658	*FOXP1*	3.708	*MYCNOS*	3.81
*ZEB2^††^*	5.513	*BMP1^†^*	4.904	*ABCC1*	4.526	*CCRL2*	3.671	*GALNT12*	3.70

Highest ranked genes are those with the smallest *P*-values and largest differences between groups. The first group forms the reference group, while the second group forms the comparison.

Overlap with genes containing differentially methylated CpGs during pregnancy in discovery^*†*^ (*n* = 21) and replication^*††*^ (*n* = 27) cohorts in Gruzieva *et al.* (2019).

The top 10 genes listed for breastfeeding did not overlap with the pregnancy-associated genes. These genes were DNAH10 (associated with gamma-delta T-cells, thought to operate at the interface between the innate and adaptive immune response), FAM193B (linked with immune tolerance to self and autoimmunity), MLNR (the motilin receptor, expressed in the gastrointestinal tract and thyroid, but not commonly found in immune cells), SLC38A10 (a sodium-coupled amino acid transport protein) and AMBRA1 (a protein involved in controlling regulatory T-cell differentiation and maintenance, and linked to tumor growth and multiple sclerosis). One gene, DNAH10 (enhanced in blood in gamma-delta and naïve CD8T cells and tied to pathways of neurodegeneration), appeared in the top 10 ranked annotated genes for both nulliparous-breastfeeding and parous-breastfeeding (Table 2). Breastfeeding networks were enriched for transmembrane transport and cell maturation (Supplementary Figs S9 and S10). A differentially methylated region in breastfeeding compared to parous and nulliparous women, spanning 2379 basepairs and covering 49 CpG sites, contained the HOXA5 and HOXA6 genes (Supplementary Fig. S12 and Table S6). These highly conserved homeobox genes are integral to embryonic development, morphogenesis and cell differentiation. HOXA5 expression has also been widely-linked to breast cancer progression. None of the top-ranked genes between nulliparous and parous women overlapped with the other comparisons between reproductive groups. However, networks comparing nulliparous and parous women were enriched for pathways tied to neuron differentiation and axonogenesis (Supplementary Fig. S11). The top differentially methylated regions for all comparisons are provided in Supplementary Table S6.

## DISCUSSION

CoR are central to evolutionary theory and supported by epidemiological patterns of disease risk that accompany parity in women. To explore tradeoffs between reproduction and immune regulation, with potential implications for health, we examined how immune cell type and regulatory processes differ between women in different reproductive states. We find evidence that immune cell composition differs by reproductive status, with a strong shift from acquired to pro-inflammatory/innate immunity during pregnancy. Most of the putative changes in cell composition seem to be resolved during and after breastfeeding. However, differences in both monocytes and NK cell proportions remain, ending up lower and higher, respectively, among breastfeeding and parous women compared to their nulliparous counterparts. There was no evidence that immune profiles differed in relation to time since parturition among post-parous women, pointing to potentially persistent changes in cell composition that accompany reproduction in women. These findings are broadly consistent with those identified using other methods [27, 28] but suggest that differences may be more persistent, highlighting potential links between the immune system and CoR in women.

### Immune cell composition with reproductive status

During pregnancy and decidualization, peripheral NK (pNK) cells migrate to the fetal–maternal interface and differentiate into uterine natural killer cells [46]. This process may reduce the relative proportion of pNK cells in pregnant women, helping to explain our observations, and may contribute to higher susceptibility of pregnant women to bacterial and viral infections [46]. In contrast, the apparent elevation in the proportion of pNK cells among breastfeeding and parous women in our study is consistent with elevated post-pregnancy immunosurveillance and ‘clearance’ of fetal-derived cells and cell-free DNA [19]. Beyond pregnancy, elevated pNK cell surveillance is protective against certain cancers, but can also increase the risk of demyelinating events that characterize multiple sclerosis [47]. To the extent that elevated pNK cells among parous women arise as a persistent effect of pregnancy, elevated pNK cell count might help explain reductions in certain cancers and elevated multiple sclerosis risk that accompany parity in women [3]. Persistent increases in pNK cells would also be consistent with long-term elevations in pro-inflammatory innate immune processes.

Monocytes are heterogeneous cell types that play a key role in pregnancy and placentation [27]. Circulating monocytes localize at the fetal–placental interface, where they are thought to be activated by contact with the syncytiotrophoblast of the placenta [27]. Locally, decidual monocytes establish immune balance between the uterus and placenta, regulating invasion of the extravillous trophoblast and remodeling of the uterine smooth muscle, glands and spiral arteries [48]. Elevated monocyte counts resulting from pregnancy have been proposed to explain the higher risk of demyelinating diseases, such as multiple sclerosis among childbearing women [49]. However, most inflammatory and autoimmune diseases that are exacerbated with parity are associated with elevated monocyte counts, and not the depressed levels we observed among breastfeeding and parous women, making the biological connection between lower monocyte counts and CoR unclear. Pro-inflammatory changes along a continuum of innate-acquired immunity may therefore contribute to long-term CoR, even if not all cell types are cleanly partitioned along these two axes.

### DNAm among pregnant women

Accounting for differences in cell composition, we also documented differences in DNAm within immune cells between women in different reproductive states. Several of our high scoring differentially methylated genes (SBNO2 and CUEDC1) are associated with body mass index (BMI)/inflammation and cervical cancer. All but one of our top-ranked genes replicate recent work by Gruzieva *et al.* [34] and suggest that changes in DNAm could be important in understanding the association between reproductive history and obesity, inflammatory disease and cervical cancer in women [3, 34]. Compared to nullipara (and to a lesser degree parous women), pregnancy was accompanied by marked hypomethylation. These findings are consistent with genomic responses to cellular and metabolic stress [50], and with decreases in DNAm and increases in gene transcription during pregnancy reported elsewhere [33–35, 51].

Hypomethylation during pregnancy may also be related to the ‘contamination’ of maternal blood with placental or fetal nucleated red blood cells (fetal microchimerism), both of which are hypomethylated compared to the other cell types examined here [52]. Supporting this hypothesis is the observation that one gene in particular (MAP3K14)—shown elsewhere to be differentially methylated between maternal and fetal nucleated red blood cells [52]—differed between pregnant and nulliparous women at 4 out of 5 probes, although none of these differences passed our false discovery threshold. Fetal microchimerism is consistent with our previous work showing younger epigenetic age among pregnant women [24], which could have long-term effects on maternal health and provide a potential source of CoR in women [19]. These findings raise the intriguing possibility of using commonly available DNAm data to infer fetal microchimerism among pregnant women, and additional work aimed at deconvoluting the contribution of placental and fetal-derived nucleated red blood cells from maternal blood epigenome is warranted.

### DNAm among breastfeeding women

The causes and consequences of global hypermethylation among breastfeeding women are less clear. Contrary to a model in which changes during pregnancy return to baseline during breastfeeding, only a small subset of the hypermethylated sites during breastfeeding overlapped with those that were hypomethylated during pregnancy. Differences in DNAm among breastfeeding women were more numerous, but also more heterogeneous than those among pregnant women, as indicated by sparser, less cohesive enrichment networks. These patterns may reflect individual heterogeneity in the frequency, duration and intensity in breastfeeding practices between women, or more developmentally contingent early life programing on lactation.

A number of the highest-ranking genes associated with breastfeeding are involved in energy storage and metabolism, consistent with the energetically taxing nature of lactation [53]. Although extrapolating our findings beyond immune cells is speculative, concordance between tissues at certain loci can be high [54]. Differences in DNAm in the GNRH2 gene during breastfeeding are consistent with the adaptive suppression of ovarian function during breastfeeding [55] and may be a target for research on the link between obesity, metabolism and infertility. Similarly, DNAH10, FAM193B and FAM13A all have robust relationships with BMI, triglyceride levels, waist-to-hip ratio, body-mass independent waist-to-hip ratio and insulin resistance [56]. FAM193B has also been linked to pronounced sex differences in adiposity, which could be especially relevant when studying the relationship between reproduction and obesity among women. Among post-reproductive women, BMI is lower among women who breastfeed compared to those who do not and decreases with time spent breastfeeding [57]. To the extent that our findings in DNAm in immune cells reflect biological processes in other tissues, the differences in DNAm described here point to the regulation of pathways of energy mobilization during breastfeeding that could affect adiposity and body mass later in life.

### DNAm among parous women

In contrast to what appear to be persistent changes in cell composition that accompany reproduction, we did not see significant differences in cell-type corrected DNAm between nulliparous and parous women at individual CpG loci after accounting for false positive rates. These findings are most consistent with transient alterations to DNAm in individual immune cells themselves during pregnancy and breastfeeding, with a return to the original methylation state among parous women. Similar findings have been reported for changes in cytokine production and gene transcription associated with pregnancy, which are largely resolved 1-year after parturition [58]. Nevertheless, persistent changes in self-recognition and immunosurveillance triggered by pregnancy and/or breastfeeding could be small, cumulative with parity and difficult to quantify given the diversity of cell types examined and individual variability in immunoregulation. Our finding that DNAm differed significantly between parous and nulliparous women for certain biological processes despite the absence of differences in individual CpGs themselves may be attributable to the power of the non-parametric method, we used for quantifying enrichment (which does not employ strict cut-offs based on *P*-value), and/or to individual heterogeneity in these women’s underlying biology and reproductive history. This model would be more consistent with historical and epidemiological evidence for CoR in women, in which the gradual erosion of regulatory processes during reproduction would manifest as small but cumulative effects on women’s health over time [2, 4].

### Limitations and future directions

We leveraged genome-wide DNAm to study the link between reproduction and women’s health from an evolutionary perspective. However, many of the differences between groups were small, with delta-betas below 0.1. One reason for these small effects may be that this subset of women is heterogeneous for numerous social and environmental exposures thought to affect DNAm, including nutrition and exposure to infectious disease and environmental pollutants [59]. Furthermore, our modest sample sizes for each group did not allow us to include parity, the duration of pregnancy, or the duration, intensity or frequency of breastfeeding at the time of sampling. This variation is likely to contribute to unmodeled variation in maternal DNAm within reproductive groups, weakening our statistical power for comparisons between groups [33, 34]. Nevertheless, all but one of our top-ranking genes replicate those found in other studies [34], supporting the robustness of our analytic approach and the findings that were present.

Another limitation is that our analysis was restricted to a cross-sectional study of young women in different reproductive states, not individual women through time. This approach limits our ability to make definitive claims about ‘changes’ in the methylome throughout reproduction, and may fail to detect small, incremental effects that accrue as women age. The use of a prospective cohort should attenuate confounding for some factors; all women were enrolled prenatally and are the same age, reducing the influence of cohort bias, participation bias or secular changes in fertility. Many of these women still differ in health and access to resources, however, which could confound our findings by affecting reproductive decisions and the methylome. For example, nulliparous women had higher SES than women in the other reproductive states, which could generate false positives in our comparisons. We addressed this statistically by including a composite measure of SES for both the year the blood sample was taken as well as the year the woman was born. We also included comparisons between pregnant or breastfeeding women and parous women, who did not differ in SES from pregnant or breastfeeding women. These two measures—combined with the fact that there were no significant individual DMPs when comparing nulliparous to parous women—support the interpretation that many of our findings are a result of differences in reproductive status and not SES. Nevertheless, a longitudinal approach, following individual women over time and through reproductive transitions, will be vital to fully address these limitations. This may be particularly important given the relatively young age of participants and narrow range of parity in our study (0–5 pregnancies), and research in higher parity women at older ages may be necessary to detect small but cumulative changes in the methylome.

This study points to changes in cell composition and DNAm that may be important for understanding the relationship between reproduction and women’s health. However, we still do not know if these differences ultimately lead to differences in health later in life. To confirm this requires longer-term studies where cell composition and DNAm during reproduction can be linked to health and aging later in life. A complementary approach would use the differentially methylated genes identified here as candidate loci for studying disease phenotypes that are linked to reproduction, such as multiple sclerosis, cancer or metabolic disorders. For example, our finding that a breastfeeding was strongly associated with a differentially methylated region that includes HOXA5, a gene tied to embryonic development and cellular differentiation, but also risk for breast cancer, provides a candidate gene linking breastfeeding and breast cancer risk that may merit further research [12]. While breast cancer and other diseases no doubt arise through a multitude of genetic and environmental factors, gaining insight into potentially modifiable genes and pathways that undergird them could shed light on the etiology of the diseases themselves or point to new diagnostic or treatment opportunities.

## CONCLUSIONS

We document widespread transient differences in immune cell composition and DNAm during pregnancy and lactation, with evidence for more modest, persistent differences immune cell composition between nulliparous and parous women. These differences may relate to evolutionarily theorized CoR in women, and broader epidemiological patterns of disease risk that accompany women’s reproductive histories. While cross-sectional and observational in nature, this study highlights the potential utility of using DNAm from the widely available Illumina microarray platform for studying the role of immunity as a potential pathway for the CoR in humans.

## Supplementary Material

eoac003_Supplementary_Data
