# Supplementary material for: Immune cell type and DNA methylation vary with reproductive status in women: possible pathways for costs of reproduction
Source: Evol Med Public Health. 2022 Feb 2;10(1):47–58. doi: 10.1093/emph/eoac003 (PMC8841013; doi:10.1093/emph/eoac003)
Supplement: eoac003_Supplementary_Data [file eoac003_supplementary_data.docx]

**Supplementary Methods**

DNA measurement and pre-processing

Overnight fasting blood samples were collected into EDTA-coated vacutainer tubes, centrifuged to separate plasma and white blood cells, and frozen at −70°C. Samples were express shipped to the US on dry ice, stored frozen at −80°C prior to DNA extraction (Puregene, Gentra), and stored at −80°C following extraction. 750ng of genomic DNA was treated with sodium bisulfite (Zymo EZDNA, Zymo Research, Irvine, CA, USA), and 160ng of converted DNA was applied to the Illumina HumanMethylation450 Bead Chip under standard conditions (Illumina Inc., San Diego, CA). Technicians were blind to any information regarding participant characteristics, and samples were randomly assigned to plate, chip, and row. Background subtraction and color correction were performed using Illumina Genome Studio with default parameters. Data were then exported into R for further analysis.

Quality control was performed as part of a larger sample to confirm participant sex and replicate status, and probes for sex chromosomes were removed from further analysis. Probes associated with known single nucleotide polymorphisms (SNPs), unreliable probes with a detection p-value above 0.01, probes with fewer than three beads contributing to signal, and those previously shown to bind to multiple genomic regions (Price et al., 2013) were also removed, leaving 434,728 probes. Data were quantile normalized using the R *lumi* package, then probe types were normalized using the SWAN method (Maksimovic, Gordon, & Oshlack, 2012). Next, plate, row, and chip batch variables were assessed using PCA and corrected using the COMBAT function in the *sva* R package (Leek, Johnson, Parker, Jaffe, & Storey, 2012). Spearman correlations for one sample run in duplicate and one sample run in quadruplicate all exceeded 0.99, confirming the efficacy of positional and batch effect corrections. Finally, proportions of blood cell types were predicted using a previously established algorithm, and variance associated with cell composition was removed using a linear regression approach (Houseman et al., 2012; Jones, Islam, Edgar, & Kobor, 2015).

*Statistical Analyses*

A total of 434,728 probes passed quality control procedures. However, many DNAm sites are largely invariable between individuals and therefore unlikely to be informative with respect to reproductive status or CoR (Mill & Heijmans, 2013; Rakyan, Down, Balding, & Beck, 2011). To concentrate our analyses on sites plausibly associated with reproductive status and to reduce the burden of multiple comparisons (Bourgon, Gentleman, & Huber, 2010), we filtered out probes for which variability in β-values between the 10th and 90th percentiles in our population was <5%. This left us with a subset of 110,631 probes, which were converted from β-values to M-values prior to statistical analyses (Du et al., 2010). For hypothesis testing, probe-wise variance was determined by fitting linear regression models and applying parametric empirical Bayes smoothing formula over the entire array dataset that passed quality control using the R bioconductor package *limma* (Ritchie et al., 2015). This approach allowed for gene-wise information borrowing to better estimate the variation for each probe. The model outcomes from the subset of variable probes described above were then extracted and corrected for multiple comparisons using the method of Benjamini and Hochberg (1995). The following contrasts were made: nulliparous-pregnant, nulliparous-breastfeeding, parous-pregnant, parous-breastfeeding, nulliparous-parous. In all cases the first group, coded as “0” in the design matrix, forms the reference group and intercept, while the second group, coded as “1”, forms the comparison group and slope.

To control for possible confounding social and environmental factors known to affect DNAm (McDade et al., 2019; Zeilinger et al., 2013) we included smoking (current smoker = 1), and a composite measure of socioeconomic status (SES) for both the year the blood sample was taken and the year the participant was born. SES was measured as a combination of income, education, and assets. Participants reported their annual income from all sources, including in-kind services, and the sale of livestock or other products by household members during the prior year, which were summed to determine total household income. Incomes were deflated to 1983 levels, and log-transformed. Participants also reported on maternal education (in years) and nine assets (coded 0, 1) that were selected to capture population-relevant aspects of social class (including electricity, televisions, refrigerators, air conditioners, tape recorder, electric fans, jeepneys, cars, and home and property ownership). In addition, house construction type (i.e., light, mixed, permanent structure) was coded as 0, 1, and 2, respectively. Thus, asset scores ranged from 0 to 11. A principal components analysis was run on log income and assets at birth (1983) and at sample collection (2005) along with maternal education in Stata (v. 14.1). The first component of variation accounted for 49% of the variation and individual scores for this component of variation were used as our measure of SES.

Finally, to control for the possible confounding influence of population stratification and some of the known impact of genetic variation on DNAm, we obtained genome-wide SNPs using the Global Screening Array (Infinium Global Screening Array-24 v2.0—Illumina). After standard SNP quality control, we performed multidimensional scaling using Euclidean distance (*cmdscale* function in R) to condense SNP variability into two components representing maximal dissimilarity in the data which were used as covariates.

*Gene Ranking, Functional Enrichment and Network Construction*

Delta betas (Δβ) used for ranking the top hits were determined from effect sizes of reproductive status from simple linear regression on untransformed β-values. Gene annotation for each probe was determined using the Illumina annotation UCSC_RefGene_Name column, resulting in some probes being associated with multiple transcripts of the same gene or multiple genes (Hansen, 2015). Using the Illumina-annotated USCS_RefGene_Name annotation we gave each gene analyzed a score. Scores were comprised of the negative log of the minimum uncorrected differential methylation p-value and the log of the absolute maximum delta beta associated with the gene. The -log10 p-value and log10 delta beta values were then standardized (value-mean/standard deviation). The average of the standardized p-value and delta beta scores was the overall score for that gene. These gene scores were used for the ranking used in Table 3 and functional enrichment ranking of gene ontology (GO) terms.

GO annotations of the 17,303 annotated genes associated with the variable 450K probes used in differential methylation analysis were used as the background list. Enrichment of GO terms in the ranked list of differentially methylated genes was tested using the receiver operator characteristic (ROC) method from ErmineJ (Gillis, Mistry, & Pavlidis, 2010). Parameters were set as follows: biological process GO terms only were included, 5–100 gene set sizes, and best scoring replicates using the standardized negative log10 p-values and standardized delta betas described above. The ROC method is based on ranking of gene scores, and enrichment for a gene set occurs when the probes in the examined genes rank higher than expected by chance. This enrichment method seeks to understand changes in the biological processes associated with reproductive status, not only the statistical significance of the probes in the individual genes that make them up. When a large number of genes, all associated with a particular biological process, rank higher than expected by chance - regardless of whether they pass statistical significance individually – ErmineJ considers that biological process itself to be significantly enriched. As a result, it is possible to observe significant enrichment even when there are no differentially methylated sites within a given gene.

Statistical significance for enrichment of a biological process is reported as false discovery rates computed using the Benjamini–Hochberg method in ErmineJ. Also calculated are the multifunctionality scores of the ontology gene sets (Gillis & Pavlidis, 2011). When investigating the related functions for each gene, genes were linked to function, expression, enhancer networks, and disease risks using openly-accessible compendia and curated databases (Fishilevich et al., 2017; Rappaport et al., 2017; Stelzer et al., 2016; Uhlén et al., 2015). Genomic features were based on UCSC genome annotations (UCSC_REFGENE_GROUP) provided by Illumina. The relationship between a CpG and repetitive DNA was derived from the overlap between the intended alignment of the probe and repetitive sequences from RepeatMasker (http://www.repeatmasker.org; RepeatMasker, Institute for Systems Biology, Seattle, WA, USA) and Price et al. (Price et al., 2013). The number of repetitive basepairs in a probe were categorized as low (<10), medium (>10 and < 40), or high (>40).

Network construction from enriched biological processes was based on the ranked ErmineJ output. First, a gmt file containing GO group terms and associated genes was created using annotation downloaded from (<http://download.baderlab.org/EM_Genesets/February_01_2018/Human/>). From this list, genesets with an adjusted p-value threshold (FDR Q value ≤ 0.1) were used to construct networks. Networks were generated using the EnrichmentMap application in Cytoscape with the additional parameters: p = 0.05, overlap coefficient 0.5 (Merico, Isserlin, Stueker, Emili, & Bader, 2010; Shannon, 2003).

Delta betas (Δβ) used for ranking the top hits were determined from effect sizes of reproductive status from simple linear regression on untransformed β-values. Gene annotation for each probe was determined using the Illumina annotation UCSC_RefGene_Name column, resulting in some probes being associated with multiple transcripts of the same gene or multiple genes (Hansen, 2015). Using the Illumina-annotated USCS_RefGene_Name annotation we gave each gene analyzed a score. Scores were comprised of the negative log of the minimum uncorrected differential methylation p-value and the log of the absolute maximum delta beta associated with the gene. The -log10 p-value and log10 delta beta values were then standardized (value-mean/standard deviation). The average of the standardized p-value and delta beta scores was the overall score for that gene. These gene scores were used for the ranking used in Table 3 and functional enrichment ranking of gene ontology (GO) terms.

GO annotations of the 17,303 annotated genes associated with the variable 450K probes used in differential methylation analysis were used as the background list. Enrichment of GO terms in the ranked list of differentially methylated genes was tested using the receiver operator characteristic (ROC) method from ErmineJ (Gillis et al., 2010). The ROC method is based on ranking of gene scores, and enrichment for a gene set occurs when the probes in the examined genes rank higher than expected by chance. Parameters were set as follows: biological process GO terms only were included, 5–100 gene set sizes, and best scoring replicates using the standardized negative log10 p-values and standardized delta betas described above. As a result, it is possible to observe significant enrichment even when there are no differentially methylated sites within a given gene. Statistical significance is reported as false discovery rates computed using the Benjamini–Hochberg method in ErmineJ. Also calculated are the multifunctionality scores of the ontology gene sets (Gillis & Pavlidis, 2011). When investigating the related functions for each gene, genes were linked to function, expression, enhancer networks, and disease risks using openly-accessible compendia and curated databases (Fishilevich et al., 2017; Rappaport et al., 2017; Stelzer et al., 2016; Uhlén et al., 2015).

Network construction from enriched biological processes was based on the ranked ErmineJ output. First, a gmt file containing GO group terms and associated genes was created using annotation downloaded from (<http://download.baderlab.org/EM_Genesets/February_01_2018/Human/>). From this list, genesets with an adjusted p-value threshold (FDR Q value ≤ 0.1) were used to construct networks. Networks were generated using the EnrichmentMap application in Cytoscape with the additional parameters: p = 0.05, overlap coefficient 0.5 (Merico et al., 2010; Shannon, 2003)

*Differentially methylated region (DMR) analysis*

DMRs were created using the ‘bumphunter’ method outlined in (Jaffe et al., 2012) and implemented using ChAMP (Morris et al., 2014). The matrix of normalized beta-values for the entire 450k array – with missing values imputed as recommended – were run using default parameters, with clusters formed using a minimum of 7 probes, the maximum gap between CpGs set to 300 basepairs, and the significance threshold for DMRs set to 0.05. Null candidate gene regions were defined using bootstrapping with 250 resamplings. For each reproductive group comparison, the top DMR was selected and presented in Table S6. The top most significant DMR – comparing breastfeeding to parous women – is also plotted in Figure S12.

**Table S1.** Differences in proportion of major immune cell-types by reproductive status, controlling for age, socioeconomic status, genetic variation, and smoking status. Significant differences are highlighted in bold.

|  | **Bcell** | | | **CD 4 T** | | | **CD 8 T** | | | **Gran** | | | **Mono** | | | **NK** | | |
| --- | --- | --- | --- | --- | --- | --- | --- | --- | --- | --- | --- | --- | --- | --- | --- | --- | --- | --- |
| *Predictors* | *Estimates* | *CI* | *p* | *Estimates* | *CI* | *p* | *Estimates* | *CI* | *p* | *Estimates* | *CI* | *p* | *Estimates* | *CI* | *p* | *Estimates* | *CI* | *p* |
| (Intercept) | 0.20 | 0.08 – 0.33 | **0.002** | 0.13 | -0.11 – 0.36 | 0.295 | 0.23 | 0.04 – 0.41 | **0.016** | 0.26 | -0.18 – 0.71 | 0.243 | 0.10 | -0.04 – 0.23 | 0.162 | 0.11 | -0.13 – 0.36 | 0.360 |
| icage05_blood | -0.01 | -0.01 – 0.00 | 0.074 | 0.00 | -0.01 – 0.01 | 0.962 | -0.01 | -0.01 – 0.00 | 0.147 | 0.01 | -0.01 – 0.03 | 0.190 | -0.00 | -0.01 – 0.00 | 0.629 | -0.00 | -0.01 – 0.01 | 0.904 |
| SES3 | 0.00 | -0.00 – 0.00 | 0.578 | 0.00 | 0.00 – 0.01 | **0.002** | 0.00 | 0.00 – 0.01 | **0.001** | -0.00 | -0.01 – 0.00 | 0.119 | -0.00 | -0.00 – 0.00 | 0.942 | -0.00 | -0.01 – -0.00 | **0.001** |
| MDS1 | 0.00 | -0.00 – 0.00 | 0.137 | 0.00 | 0.00 – 0.00 | **0.021** | -0.00 | -0.00 – -0.00 | **<0.001** | 0.00 | -0.00 – 0.00 | 0.389 | -0.00 | -0.00 – 0.00 | 0.120 | -0.00 | -0.00 – 0.00 | 0.298 |
| MDS2 | 0.00 | -0.00 – 0.00 | 0.845 | 0.00 | -0.00 – 0.00 | 0.314 | 0.00 | -0.00 – 0.00 | 0.134 | 0.00 | -0.00 – 0.00 | 0.707 | -0.00 | -0.00 – 0.00 | 0.447 | -0.00 | -0.00 – -0.00 | **0.009** |
| smoker | 0.01 | -0.01 – 0.02 | 0.384 | 0.01 | -0.01 – 0.04 | 0.274 | 0.00 | -0.01 – 0.02 | 0.604 | -0.00 | -0.05 – 0.04 | 0.909 | -0.00 | -0.01 – 0.01 | 0.970 | -0.02 | -0.05 – 0.00 | 0.099 |
| reprostat [Pregnant] | -0.02 | -0.02 – -0.01 | **<0.001** | -0.03 | -0.04 – -0.02 | **<0.001** | -0.01 | -0.02 – -0.00 | **0.006** | 0.08 | 0.06 – 0.10 | **<0.001** | 0.01 | 0.01 – 0.02 | **<0.001** | -0.04 | -0.05 – -0.02 | **<0.001** |
| reprostat [Breastfeeding] | -0.00 | -0.01 – 0.00 | 0.558 | -0.00 | -0.01 – 0.01 | 0.689 | 0.01 | -0.00 – 0.02 | 0.074 | -0.00 | -0.02 – 0.02 | 0.812 | -0.01 | -0.02 – -0.00 | **0.001** | 0.01 | -0.00 – 0.02 | 0.156 |
| reprostat [Parous] | -0.01 | -0.01 – 0.00 | 0.052 | -0.00 | -0.01 – 0.01 | 0.587 | 0.00 | -0.01 – 0.01 | 0.708 | 0.00 | -0.01 – 0.02 | 0.609 | -0.01 | -0.02 – -0.00 | **0.001** | 0.01 | 0.00 – 0.02 | **0.032** |
| Observations | 392 | | | 392 | | | 392 | | | 392 | | | 392 | | | 392 | | |
| R^2^ / R^2^ adjusted | 0.106 / 0.088 | | | 0.142 / 0.124 | | | 0.105 / 0.086 | | | 0.180 / 0.163 | | | 0.134 / 0.116 | | | 0.175 / 0.158 | | |

**Table S2.** Differences in proportion of major immune cell-types by reproductive status, controlling for age, socioeconomic status, genetic variation, and smoking status, with “parous” as the reference level. Significant differences are highlighted in bold.

|  | **Bcell** | | | **CD 4 T** | | | **CD 8 T** | | | **Gran** | | | **Mono** | | | **NK** | | |
| --- | --- | --- | --- | --- | --- | --- | --- | --- | --- | --- | --- | --- | --- | --- | --- | --- | --- | --- |
| *Predictors* | *Estimates* | *CI* | *p* | *Estimates* | *CI* | *p* | *Estimates* | *CI* | *p* | *Estimates* | *CI* | *p* | *Estimates* | *CI* | *p* | *Estimates* | *CI* | *p* |
| (Intercept) | 0.20 | 0.07 – 0.32 | **0.002** | 0.12 | -0.11 – 0.36 | 0.307 | 0.23 | 0.04 – 0.41 | **0.016** | 0.27 | -0.18 – 0.71 | 0.235 | 0.09 | -0.05 – 0.22 | 0.212 | 0.12 | -0.12 – 0.37 | 0.316 |
| icage05_blood | -0.01 | -0.01 – 0.00 | 0.074 | 0.00 | -0.01 – 0.01 | 0.962 | -0.01 | -0.01 – 0.00 | 0.147 | 0.01 | -0.01 – 0.03 | 0.190 | -0.00 | -0.01 – 0.00 | 0.629 | -0.00 | -0.01 – 0.01 | 0.904 |
| SES3 | 0.00 | -0.00 – 0.00 | 0.578 | 0.00 | 0.00 – 0.01 | **0.002** | 0.00 | 0.00 – 0.01 | **0.001** | -0.00 | -0.01 – 0.00 | 0.119 | -0.00 | -0.00 – 0.00 | 0.942 | -0.00 | -0.01 – -0.00 | **0.001** |
| MDS1 | 0.00 | -0.00 – 0.00 | 0.137 | 0.00 | 0.00 – 0.00 | **0.021** | -0.00 | -0.00 – -0.00 | **<0.001** | 0.00 | -0.00 – 0.00 | 0.389 | -0.00 | -0.00 – 0.00 | 0.120 | -0.00 | -0.00 – 0.00 | 0.298 |
| MDS2 | 0.00 | -0.00 – 0.00 | 0.845 | 0.00 | -0.00 – 0.00 | 0.314 | 0.00 | -0.00 – 0.00 | 0.134 | 0.00 | -0.00 – 0.00 | 0.707 | -0.00 | -0.00 – 0.00 | 0.447 | -0.00 | -0.00 – -0.00 | **0.009** |
| smoker | 0.01 | -0.01 – 0.02 | 0.384 | 0.01 | -0.01 – 0.04 | 0.274 | 0.00 | -0.01 – 0.02 | 0.604 | -0.00 | -0.05 – 0.04 | 0.909 | -0.00 | -0.01 – 0.01 | 0.970 | -0.02 | -0.05 – 0.00 | 0.099 |
| reprostat [Nulliparous] | 0.01 | -0.00 – 0.01 | 0.052 | 0.00 | -0.01 – 0.01 | 0.587 | -0.00 | -0.01 – 0.01 | 0.708 | -0.00 | -0.02 – 0.01 | 0.609 | 0.01 | 0.00 – 0.02 | **0.001** | -0.01 | -0.02 – -0.00 | **0.032** |
| reprostat [Pregnant] | -0.01 | -0.02 – -0.01 | **<0.001** | -0.03 | -0.04 – -0.02 | **<0.001** | -0.01 | -0.02 – -0.00 | **0.007** | 0.08 | 0.06 – 0.10 | **<0.001** | 0.02 | 0.02 – 0.03 | **<0.001** | -0.05 | -0.06 – -0.03 | **<0.001** |
| reprostat [Breastfeeding] | 0.00 | -0.00 – 0.01 | 0.313 | 0.00 | -0.01 – 0.01 | 0.943 | 0.01 | -0.00 – 0.02 | 0.197 | -0.01 | -0.03 – 0.02 | 0.541 | -0.00 | -0.01 – 0.01 | 0.851 | -0.00 | -0.02 – 0.01 | 0.670 |
| Observations | 392 | | | 392 | | | 392 | | | 392 | | | 392 | | | 392 | | |
| R^2^ / R^2^ adjusted | 0.106 / 0.088 | | | 0.142 / 0.124 | | | 0.105 / 0.086 | | | 0.180 / 0.163 | | | 0.134 / 0.116 | | | 0.175 / 0.158 | | |


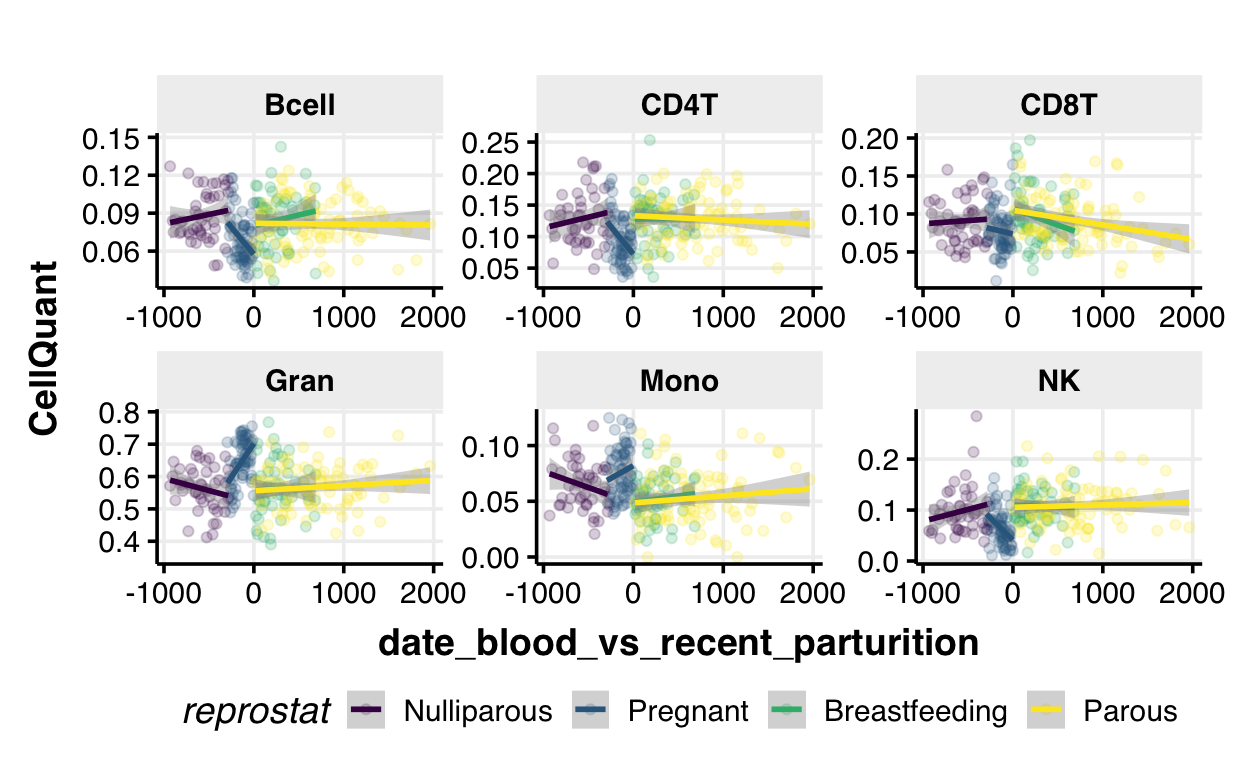


**Figure S1.** Plot of cell composition relative to end of pregnancy (live birth, stillbirth, miscarriage) for different reproductive status groups (nulliparous, pregnant, breastfeeding, and parous, but not pregnant or breastfeeding).

Table S3. Differences in proportion of major immune cell-types with days after the end of pregnancy for parous women, controlling for age, socioeconomic status, genetic variation, and smoking status. Significant differences are highlighted in bold.

|  | **Bcell** | | | **CD 4 T** | | | **CD 8 T** | | | **Gran** | | | **Mono** | | | **NK** | | |
| --- | --- | --- | --- | --- | --- | --- | --- | --- | --- | --- | --- | --- | --- | --- | --- | --- | --- | --- |
| *Predictors* | *Estimates* | *CI* | *p* | *Estimates* | *CI* | *p* | *Estimates* | *CI* | *p* | *Estimates* | *CI* | *p* | *Estimates* | *CI* | *p* | *Estimates* | *CI* | *p* |
| (Intercept) | 0.32 | 0.07 – 0.57 | **0.012** | 0.08 | -0.38 – 0.53 | 0.741 | 0.20 | -0.20 – 0.60 | 0.322 | 0.06 | -0.81 – 0.92 | 0.898 | 0.23 | -0.10 – 0.55 | 0.173 | 0.14 | -0.40 – 0.68 | 0.600 |
| icage05_blood | -0.01 | -0.02 – 0.00 | 0.058 | 0.00 | -0.02 – 0.02 | 0.805 | -0.00 | -0.02 – 0.01 | 0.634 | 0.02 | -0.02 – 0.06 | 0.254 | -0.01 | -0.02 – 0.01 | 0.286 | -0.00 | -0.03 – 0.02 | 0.895 |
| SES3 | -0.00 | -0.00 – 0.00 | 0.297 | 0.00 | -0.00 – 0.01 | 0.260 | 0.00 | -0.00 – 0.01 | 0.152 | -0.01 | -0.02 – 0.00 | 0.270 | 0.00 | -0.00 – 0.01 | 0.220 | -0.00 | -0.01 – 0.00 | 0.305 |
| MDS1 | -0.00 | -0.00 – 0.00 | 0.160 | 0.00 | -0.00 – 0.00 | 0.978 | -0.00 | -0.00 – 0.00 | 0.702 | 0.00 | -0.00 – 0.00 | 0.582 | 0.00 | -0.00 – 0.00 | 0.815 | -0.00 | -0.00 – 0.00 | 0.855 |
| MDS2 | 0.00 | -0.00 – 0.00 | 0.802 | 0.00 | -0.00 – 0.00 | 0.106 | 0.00 | -0.00 – 0.00 | 0.292 | -0.00 | -0.00 – 0.00 | 0.761 | -0.00 | -0.00 – 0.00 | 0.217 | -0.00 | -0.00 – 0.00 | 0.312 |
| smoker | 0.00 | -0.01 – 0.02 | 0.686 | 0.02 | -0.00 – 0.05 | 0.103 | 0.01 | -0.02 – 0.03 | 0.490 | -0.01 | -0.06 – 0.04 | 0.672 | 0.00 | -0.02 – 0.02 | 0.947 | -0.03 | -0.06 – 0.01 | 0.126 |
| date_blood_vs_recent_parturition | -0.00 | -0.00 – 0.00 | 0.998 | -0.00 | -0.00 – 0.00 | 0.411 | -0.00 | -0.00 – -0.00 | **0.013** | 0.00 | -0.00 – 0.00 | 0.397 | 0.00 | -0.00 – 0.00 | 0.226 | 0.00 | -0.00 – 0.00 | 0.643 |
| Observations | 87 | | | 87 | | | 87 | | | 87 | | | 87 | | | 87 | | |
| R^2^ / R^2^ adjusted | 0.076 / 0.007 | | | 0.089 / 0.021 | | | 0.122 / 0.056 | | | 0.049 / -0.022 | | | 0.061 / -0.009 | | | 0.059 / -0.012 | | |

Table S4. Differences in proportion of major immune cell-types with days after the end of pregnancy for parous women, including a polynomial term for a non-linear effect of time. All models control for age, socioeconomic status, genetic variation, and smoking status. Significant differences are highlighted in bold.

|  | **Bcell** | | | **CD 4 T** | | | **CD 8 T** | | | **Gran** | | | **Mono** | | | **NK** | | |
| --- | --- | --- | --- | --- | --- | --- | --- | --- | --- | --- | --- | --- | --- | --- | --- | --- | --- | --- |
| *Predictors* | *Estimates* | *CI* | *p* | *Estimates* | *CI* | *p* | *Estimates* | *CI* | *p* | *Estimates* | *CI* | *p* | *Estimates* | *CI* | *p* | *Estimates* | *CI* | *p* |
| (Intercept) | 0.31 | 0.06 – 0.56 | **0.015** | 0.05 | -0.40 – 0.49 | 0.839 | 0.18 | -0.22 – 0.59 | 0.374 | 0.09 | -0.79 – 0.96 | 0.845 | 0.25 | -0.08 – 0.57 | 0.134 | 0.15 | -0.39 – 0.70 | 0.582 |
| icage05_blood | -0.01 | -0.02 – 0.00 | 0.069 | 0.00 | -0.02 – 0.02 | 0.722 | -0.00 | -0.02 – 0.01 | 0.658 | 0.02 | -0.02 – 0.06 | 0.276 | -0.01 | -0.02 – 0.01 | 0.241 | -0.00 | -0.03 – 0.02 | 0.881 |
| SES3 | -0.00 | -0.00 – 0.00 | 0.217 | 0.00 | -0.00 – 0.01 | 0.359 | 0.00 | -0.00 – 0.01 | 0.176 | -0.01 | -0.02 – 0.01 | 0.318 | 0.00 | -0.00 – 0.01 | 0.155 | -0.00 | -0.01 – 0.00 | 0.331 |
| MDS1 | -0.00 | -0.00 – 0.00 | 0.163 | 0.00 | -0.00 – 0.00 | 0.958 | -0.00 | -0.00 – 0.00 | 0.709 | 0.00 | -0.00 – 0.00 | 0.590 | 0.00 | -0.00 – 0.00 | 0.832 | -0.00 | -0.00 – 0.00 | 0.852 |
| MDS2 | 0.00 | -0.00 – 0.00 | 0.553 | 0.00 | 0.00 – 0.00 | **0.049** | 0.00 | -0.00 – 0.00 | 0.259 | -0.00 | -0.00 – 0.00 | 0.645 | -0.00 | -0.00 – 0.00 | 0.118 | -0.00 | -0.00 – 0.00 | 0.294 |
| smoker | 0.00 | -0.01 – 0.02 | 0.566 | 0.03 | -0.00 – 0.05 | 0.068 | 0.01 | -0.02 – 0.03 | 0.462 | -0.01 | -0.07 – 0.04 | 0.616 | -0.00 | -0.02 – 0.02 | 0.915 | -0.03 | -0.06 – 0.01 | 0.122 |
| date_blood_vs_recent_parturition [1st degree] | -0.00 | -0.04 – 0.04 | 0.980 | -0.03 | -0.10 – 0.04 | 0.391 | -0.08 | -0.14 – -0.02 | **0.014** | 0.06 | -0.08 – 0.19 | 0.393 | 0.03 | -0.02 – 0.08 | 0.213 | 0.02 | -0.06 – 0.10 | 0.641 |
| date_blood_vs_recent_parturition [2nd degree] | -0.03 | -0.07 – 0.01 | 0.125 | -0.06 | -0.13 – 0.01 | 0.087 | -0.02 | -0.08 – 0.05 | 0.629 | 0.05 | -0.09 – 0.19 | 0.461 | 0.04 | -0.01 – 0.09 | 0.116 | 0.01 | -0.07 – 0.10 | 0.754 |
| Observations | 87 | | | 87 | | | 87 | | | 87 | | | 87 | | | 87 | | |
| R^2^ / R^2^ adjusted | 0.103 / 0.024 | | | 0.122 / 0.045 | | | 0.125 / 0.047 | | | 0.056 / -0.028 | | | 0.090 / 0.010 | | | 0.060 / -0.024 | | |

**Table S5.** Number of differentially methylated positions (DMPs) among women varying in reproductive status. Numbers are in contrast to the reference (top) level (i.e. Null = nulliparous as reference group).

|  | Null-Pregnant | Parous-Pregnant | Null-Breastfeeding | Parous-Breastfeeding | Null-Parous |
| --- | --- | --- | --- | --- | --- |
| Down | 795 | 533 | 320 | 0 | 0 |
| Non-sign. | 109803 | 110092 | 109524 | 110630 | 110631 |
| Up | 33 | 6 | 787 | 1 | 0 |

**probe UCSC_REFGENE_NAME UCSC_REFGENE_GROUP P.Value adj.P.Val Dbeta**

1 cg27138901 CLEC2D;CLEC2D 3'UTR;3'UTR 4.194601e-21 4.640529e-16 -0.06353119

2 cg16936953 TMEM49 Body 3.881106e-11 3.578088e-07 -0.05263288

3 cg13108304 MCF2L;MCF2L Body;Body 1.444053e-04 2.718919e-02 0.05131485

4 cg06098276 TK1 Body 8.413607e-06 4.390593e-03 -0.05004203

Figure S2. DNAm for Top-4 CpGs comparing nulliparous to pregnant women (including all other reproductive groups), ranked by delta-β. Illumina probe id, UCSC reference genome gene name, UCSC reference genome feature group, p-value, FDR adjusted p-value, and delta-β for each probe are shown below the figure.

**probe UCSC_REFGENE_NAME UCSC_REFGENE_GROUP P.Value adj.P.Val Dbeta**

1 cg18106582 SNX18;SNX18;SNX18;SNX18 1stExon;Body;3'UTR;Body 7.940232e-05 0.016267329 0.04624372

2 cg19968199 DYRK2;DYRK2 3'UTR;3'UTR 3.290398e-05 0.009529321 -0.05550657

3 cg25301532 KCNK15 Body 3.414546e-04 0.039669929 0.05207311

4 cg26562263 <NA> <NA> 4.706522e-04 0.048346072 -0.05025309

Figure S3. DNAm for Top-4 CpGs comparing nulliparous to breastfeeding women (including all other reproductive groups), ranked by delta-β. Illumina probe id, UCSC reference genome gene name, UCSC reference genome feature group, p-value, FDR adjusted p-value, and delta-β for each probe are shown below the figure.

**probe UCSC_REFGENE_NAME UCSC_REFGENE_GROUP P.Value adj.P.Val Dbeta**

1 cg09976142 CIZ1;CIZ1 5'UTR;5'UTR 0.0005352286 0.07631132 -0.07746625

2 cg10071929 CIZ1;CIZ1;CIZ1;CIZ1;CIZ1 TSS1500;5'UTR;TSS1500;5'UTR;TSS1500 0.0005904123 0.08044076 -0.07461911

3 cg14983838 <NA> <NA> 0.0167977102 0.36756096 -0.06446171

4 cg24853868 <NA> <NA> 0.0076371811 0.27513769 -0.07613729

Figure S4. DNAm for Top-4 CpGs comparing parous to pregnant women (including all other reproductive groups), ranked by delta-β. Illumina probe id, UCSC reference genome gene name, UCSC reference genome feature group, p-value, FDR adjusted p-value, and delta-β for each probe are shown below the figure.

**probe UCSC_REFGENE_NAME UCSC_REFGENE_GROUP P.Value adj.P.Val Dbeta**

1 cg05209514 CPM 5'UTR 1.089591e-06 0.06027127 0.02765853

2 cg07549715 GNRH2;GNRH2;GNRH2 TSS200;TSS200;TSS200 1.216034e-07 0.01345311 0.02391630

3 cg14786790 FAM13A Body 2.093358e-06 0.07719676 0.02349108

4 cg18599081 CCRL2;CCRL2 TSS1500;TSS1500 3.859520e-05 0.38816599 0.02248950

Figure S5. DNAm for Top-4 CpGs comparing parous to breastfeeding women (including all other reproductive groups), ranked by delta-β. Illumina probe id, UCSC reference genome gene name, UCSC reference genome feature group, p-value, FDR adjusted p-value, and delta-β for each probe are shown below the figure.

**probe UCSC_REFGENE_NAME UCSC_REFGENE_GROUP P.Value adj.P.Val Dbeta**

1 cg03274391 <NA> <NA> 0.0004778213 0.3719536 -0.03176896

2 cg18650626 MAD1L1;MAD1L1;MAD1L1 Body;Body;Body 0.0005006797 0.3772734 -0.03017394

3 cg25105522 MAP3K14 Body 0.0001884602 0.3265828 -0.03195267

4 cg25376651 EEF1DP3 Body 0.0001529578 0.3265828 0.02944929

Figure S6. DNAm for Top-4 CpGs comparing nulliparous to parous women (including all other reproductive groups), ranked by delta-β. Illumina probe id, UCSC reference genome gene name, UCSC reference genome feature group, p-value, FDR adjusted p-value, and delta-β for each probe are shown below the figure


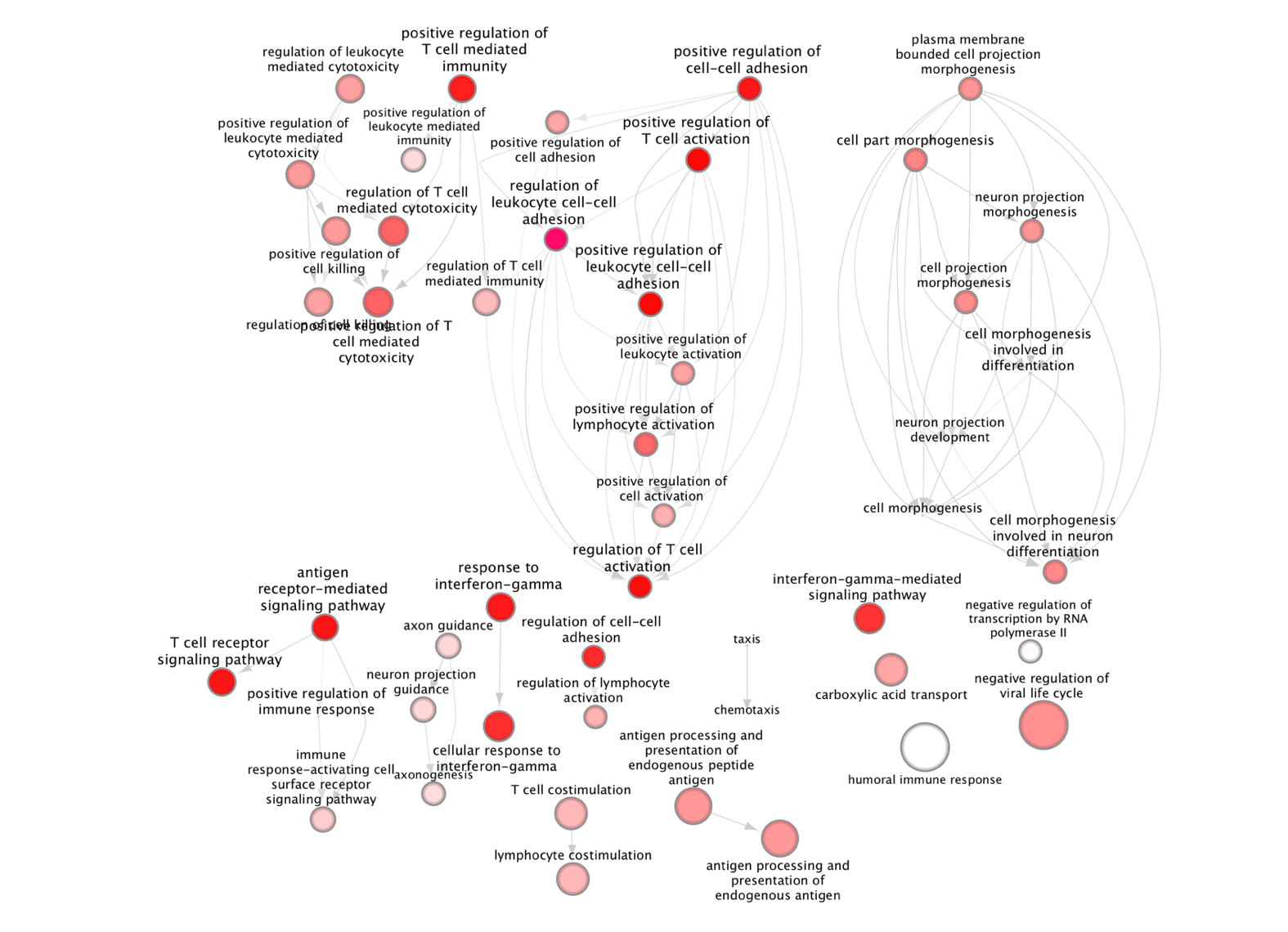


**Figure S7.** Nulliparous – Pregnant Women: Network of enriched biological processes based on differential methylation in pregnant women compared to nulliparous women. Darker colors indicate smaller false-discovery rate (FDR) corrected enrichment p-values based on ranking using the receiver operating characteristic and gene scores. Nulliparous women form the reference group. More description in the methods.


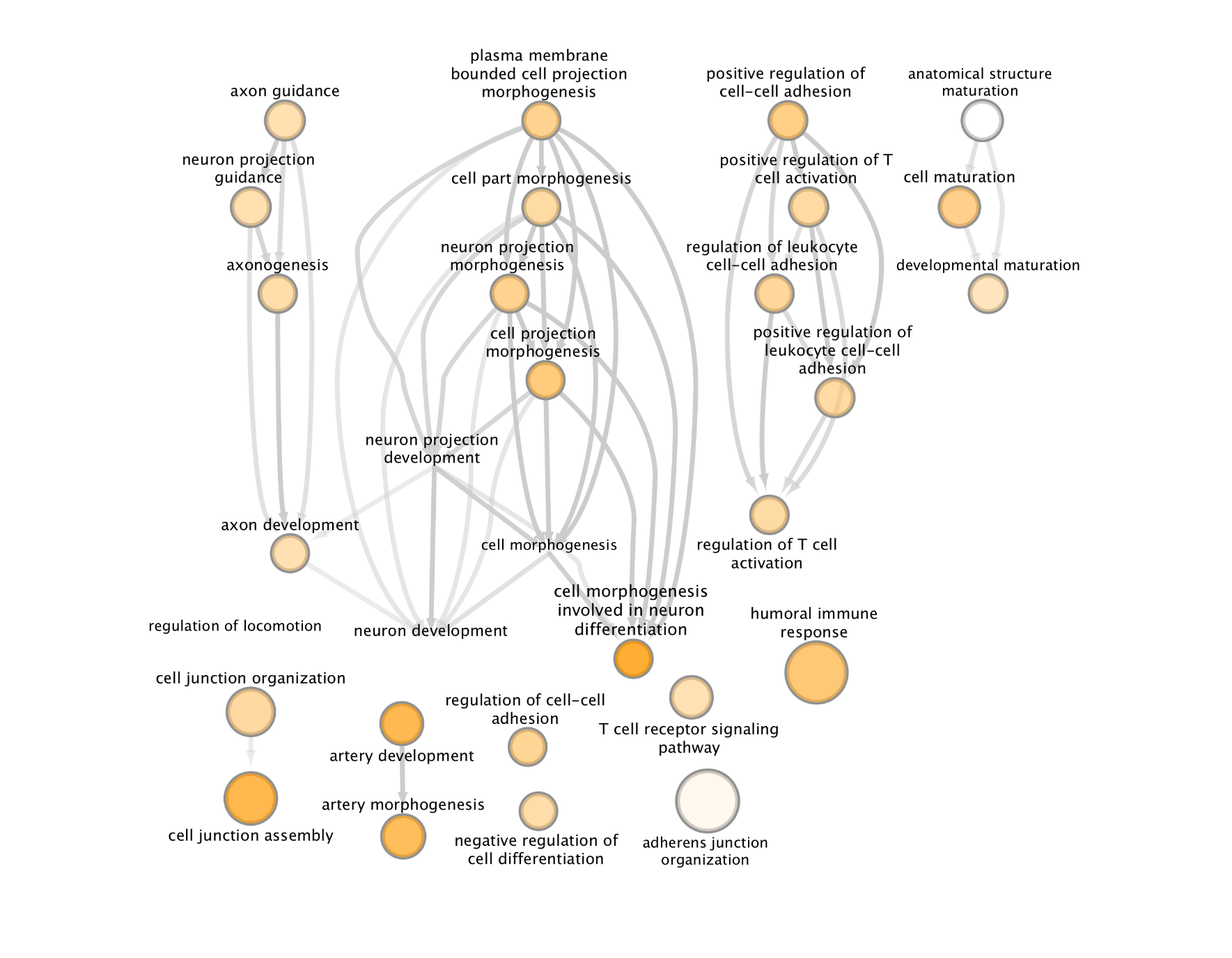


**Figure S8.** Parous – Pregnant women: Network of enriched biological processes based on differential methylation in pregnant women compared to parous women. Darker colors indicate smaller false-discovery rate (FDR) corrected enrichment p-values based on ranking using the receiver operating characteristic and gene scores. Parous women form the reference group. More description in the methods.


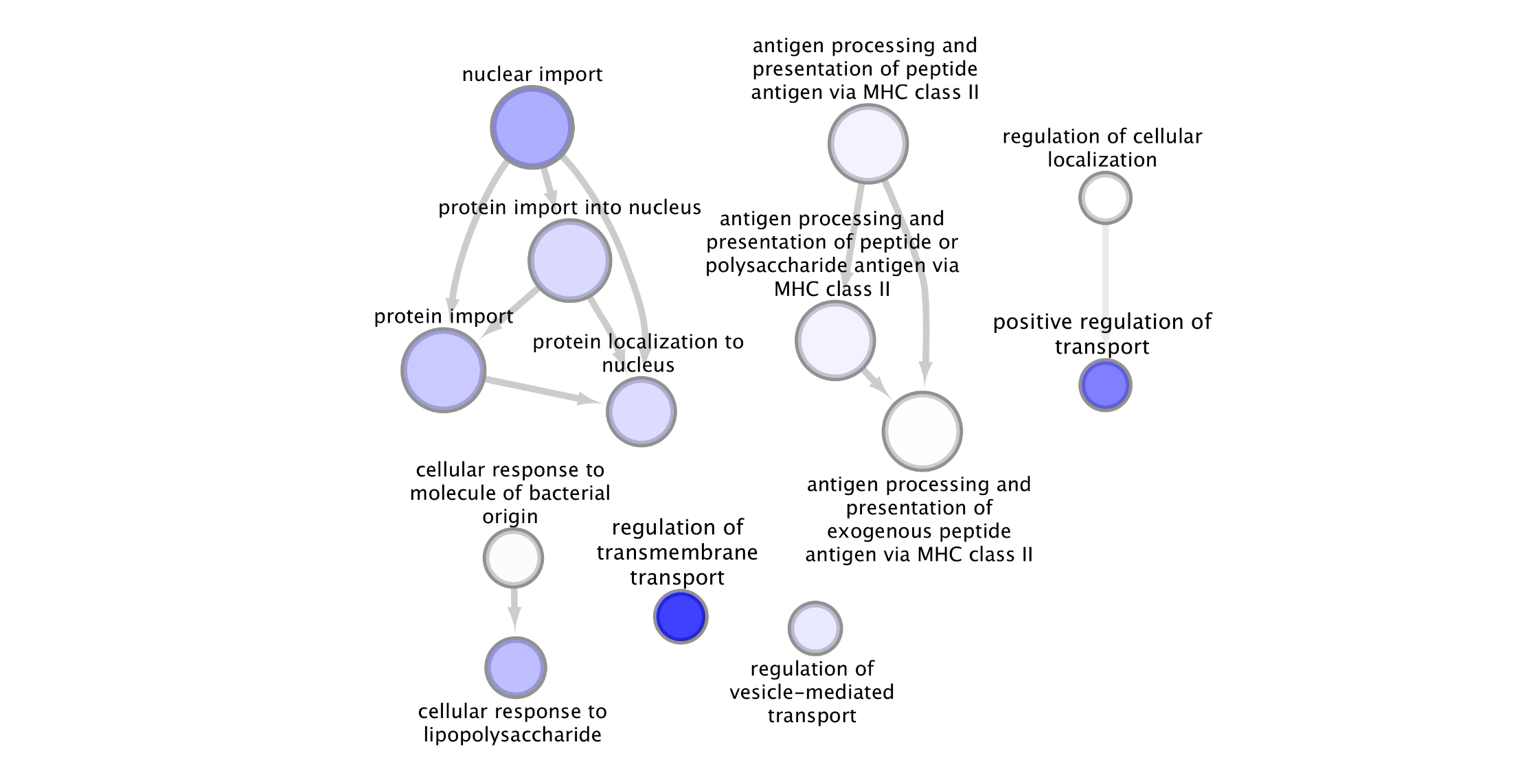


**Figure S9.** Nulliparous – Breastfeeding women: Network of enriched biological processes based on differential methylation in breastfeeding women compared to nulliparous women. Darker colors indicate smaller false-discovery rate (FDR) corrected enrichment p-values based on ranking using the receiver operating characteristic and gene scores. Nulliparous women form the reference group. More description in the methods.

**Figure S10.** Parous – Breastfeeding women: Network of enriched biological processes based on differential methylation in breastfeeding women compared to parous women. Darker colors indicate smaller false-discovery rate (FDR) corrected enrichment p-values based on ranking using the receiver operating characteristic and gene scores. Parous women form the reference group. More description in the methods.


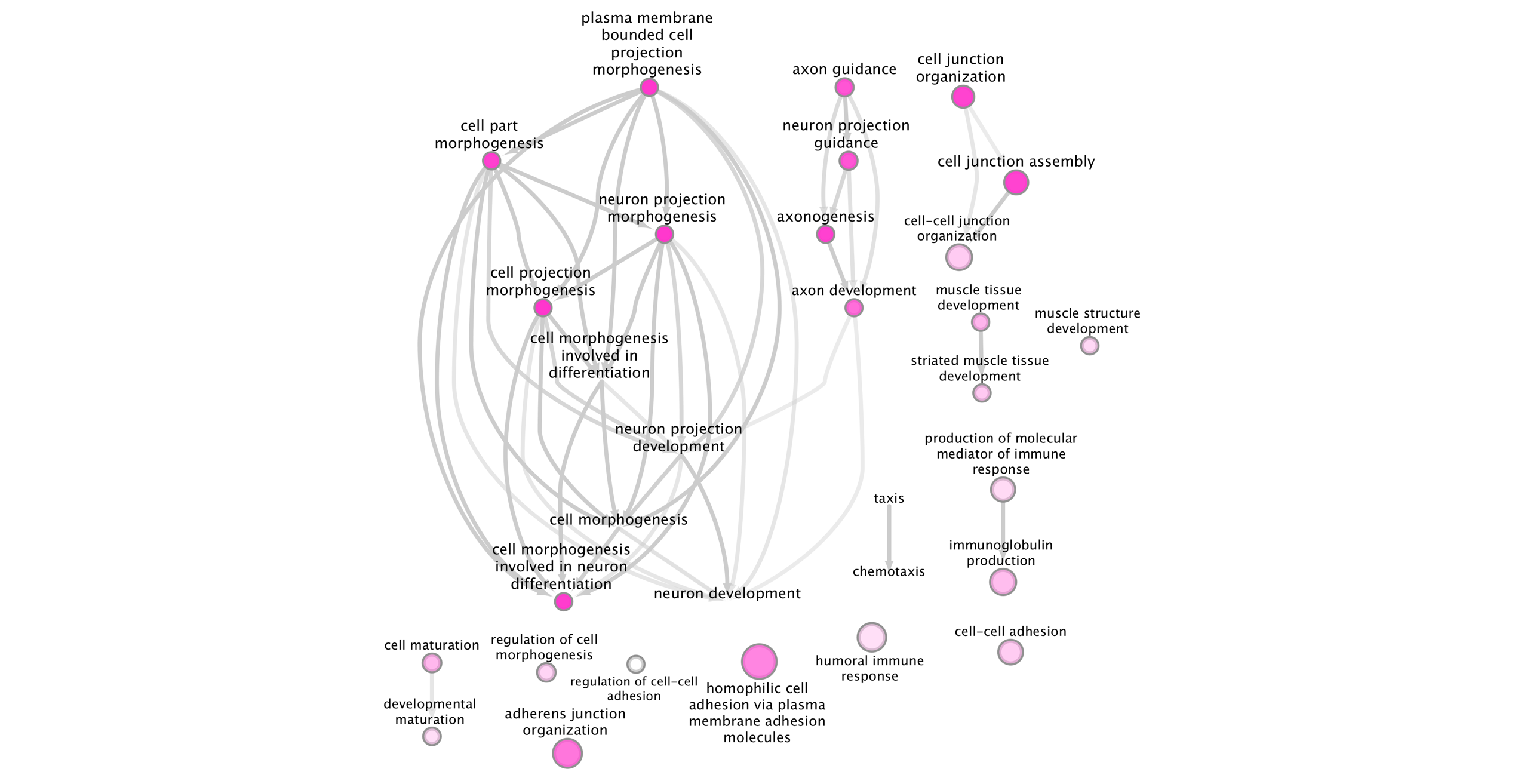


**Figure S11.** Nulliparous – Parous women: Network of enriched biological processes based on differential methylation in parous women compared to nulliparous women. Darker colors indicate smaller false-discovery rate (FDR) corrected enrichment p-values based on ranking using the receiver operating characteristic and gene scores. Nulliparous women form the reference group. More description in the methods.

Figure S12. Sample differentially methylated region (DMR) comparing breastfeeding to parous women (p-value = 5.76E-05 family-wise error rate = 0.024). The region, located between x and y on chromosome 7, spanned 2379 basepairs and covered 49 CpG sites, including HOXA5 and HOXA6 genes. Genes, CpG sites on the array, and DNAm beta-values (truncated axes) and means for each reproductive status group are shown. The top DMR for each reproductive group comparison and associated probes and gene information are provided in Table S6.

Table S6. Top ranking differentially methylated region (DMR) for each reproductive status group comparison. For each DMR, probe, chromosome (CHR), basepair number (MAPINFO), closest transcription start site gene name (Closest_TSS_gene_name), UCSC gene the probe is located in (UCSC_REFGENE_NAME), p-value for the DMR, and family-wise error rate for the DMR (fwer). All mapping based on Genome Reference Consortium Human Build 37 (GRCh37). UCSC_REFGENE_NAME listed multiple times if a probe appears in >1 transcript or gene.

| **probe** | **CHR** | **MAPINFO** | **Closest_TSS _gene_name** | **UCSC _REFGENE_NAME** | **Comparison (reference/comparison)** | **p.value** | **fwer** |
| --- | --- | --- | --- | --- | --- | --- | --- |
| cg02248486 | 7 | 27183196 | HOXA5 | HOXA5 | parous/breastfeeding | 5.76E-05 | 0.024 |
| cg25866143 | 7 | 27183262 | HOXA5 | HOXA5;HOXA5 | parous/breastfeeding | 5.76E-05 | 0.024 |
| cg09549073 | 7 | 27183274 | HOXA5 | HOXA5;HOXA5 | parous/breastfeeding | 5.76E-05 | 0.024 |
| cg01370449 | 7 | 27183369 | HOXA5 | HOXA5 | parous/breastfeeding | 5.76E-05 | 0.024 |
| cg04863892 | 7 | 27183375 | HOXA5 | HOXA5 | parous/breastfeeding | 5.76E-05 | 0.024 |
| cg19759481 | 7 | 27183401 | HOXA5 | HOXA5 | parous/breastfeeding | 5.76E-05 | 0.024 |
| cg12128839 | 7 | 27183436 | HOXA5 | HOXA5 | parous/breastfeeding | 5.76E-05 | 0.024 |
| cg02916332 | 7 | 27183591 | HOXA5 | HOXA5 | parous/breastfeeding | 5.76E-05 | 0.024 |
| cg17569124 | 7 | 27183643 | HOXA5 | HOXA5 | parous/breastfeeding | 5.76E-05 | 0.024 |
| cg02005600 | 7 | 27183686 | HOXA5 | HOXA5 | parous/breastfeeding | 5.76E-05 | 0.024 |
| cg25307665 | 7 | 27183694 | HOXA5 | HOXA5 | parous/breastfeeding | 5.76E-05 | 0.024 |
| cg14014955 | 7 | 27183701 | HOXA5 | HOXA5 | parous/breastfeeding | 5.76E-05 | 0.024 |
| cg02646423 | 7 | 27183794 | HOXA5 | HOXA5 | parous/breastfeeding | 5.76E-05 | 0.024 |
| cg20517050 | 7 | 27183806 | HOXA5 | HOXA5 | parous/breastfeeding | 5.76E-05 | 0.024 |
| cg23204968 | 7 | 27183816 | HOXA5 | HOXA5 | parous/breastfeeding | 5.76E-05 | 0.024 |
| cg05835726 | 7 | 27183861 | HOXA5 | HOXA5 | parous/breastfeeding | 5.76E-05 | 0.024 |
| cg14058329 | 7 | 27183946 | HOXA5 | HOXA5 | parous/breastfeeding | 5.76E-05 | 0.024 |
| cg03207666 | 7 | 27183950 | HOXA5 | HOXA5 | parous/breastfeeding | 5.76E-05 | 0.024 |
| cg13512268 | 7 | 27183973 | HOXA5 | HOXA5 | parous/breastfeeding | 5.76E-05 | 0.024 |
| cg23454797 | 7 | 27183990 | HOXA5 | HOXA5 | parous/breastfeeding | 5.76E-05 | 0.024 |
| cg12015737 | 7 | 27184030 | HOXA5 | HOXA5 | parous/breastfeeding | 5.76E-05 | 0.024 |
| cg08070327 | 7 | 27184059 | HOXA5 | HOXA5 | parous/breastfeeding | 5.76E-05 | 0.024 |
| cg25506432 | 7 | 27184065 | HOXA5 | HOXA5 | parous/breastfeeding | 5.76E-05 | 0.024 |
| cg14658493 | 7 | 27184077 | HOXA5 | HOXA5 | parous/breastfeeding | 5.76E-05 | 0.024 |
| cg19196335 | 7 | 27184094 | HOXA5 | HOXA5 | parous/breastfeeding | 5.76E-05 | 0.024 |
| cg09880291 | 7 | 27184109 | HOXA5 | HOXA5 | parous/breastfeeding | 5.76E-05 | 0.024 |
| cg24389585 | 7 | 27184125 | HOXA5 | HOXA5 | parous/breastfeeding | 5.76E-05 | 0.024 |
| cg16997642 | 7 | 27184159 | HOXA5 | HOXA5 | parous/breastfeeding | 5.76E-05 | 0.024 |
| cg20817131 | 7 | 27184167 | HOXA5 | HOXA5 | parous/breastfeeding | 5.76E-05 | 0.024 |
| cg14013695 | 7 | 27184176 | HOXA5 | HOXA5 | parous/breastfeeding | 5.76E-05 | 0.024 |
| cg25390165 | 7 | 27184188 | HOXA5 | HOXA5 | parous/breastfeeding | 5.76E-05 | 0.024 |
| cg01323381 | 7 | 27184264 | HOXA5 | HOXA5 | parous/breastfeeding | 5.76E-05 | 0.024 |
| cg19643053 | 7 | 27184271 | HOXA5 | HOXA5 | parous/breastfeeding | 5.76E-05 | 0.024 |
| cg05774699 | 7 | 27184316 | HOXA5 | HOXA5 | parous/breastfeeding | 5.76E-05 | 0.024 |
| cg26023912 | 7 | 27184369 | HOXA5 | HOXA5 | parous/breastfeeding | 5.76E-05 | 0.024 |
| cg14882265 | 7 | 27184375 | HOXA5 | HOXA5 | parous/breastfeeding | 5.76E-05 | 0.024 |
| cg17432857 | 7 | 27184438 | HOXA5 | HOXA5 | parous/breastfeeding | 5.76E-05 | 0.024 |
| cg00969405 | 7 | 27184441 | HOXA5 | HOXA5 | parous/breastfeeding | 5.76E-05 | 0.024 |
| cg07049592 | 7 | 27184450 | HOXA5 | HOXA5 | parous/breastfeeding | 5.76E-05 | 0.024 |
| cg02106682 | 7 | 27184461 | HOXA5 | HOXA5 | parous/breastfeeding | 5.76E-05 | 0.024 |
| cg03368099 | 7 | 27184521 | HOXA5 | HOXA5 | parous/breastfeeding | 5.76E-05 | 0.024 |
| cg01748892 | 7 | 27184667 | HOXA5 | HOXA5 | parous/breastfeeding | 5.76E-05 | 0.024 |
| cg13694927 | 7 | 27184712 | HOXA5 | HOXA5 | parous/breastfeeding | 5.76E-05 | 0.024 |
| cg03744763 | 7 | 27184737 | HOXA5 | HOXA5 | parous/breastfeeding | 5.76E-05 | 0.024 |
| cg27151303 | 7 | 27184821 | HOXA5 | NA | parous/breastfeeding | 5.76E-05 | 0.024 |
| cg05579037 | 7 | 27184853 | HOXA5 | NA | parous/breastfeeding | 5.76E-05 | 0.024 |
| cg21641458 | 7 | 27185136 | LOC100133311 | HOXA6 | parous/breastfeeding | 5.76E-05 | 0.024 |
| cg09343092 | 7 | 27185282 | LOC100133311 | HOXA6 | parous/breastfeeding | 5.76E-05 | 0.024 |
| cg18931036 | 7 | 27185393 | LOC100133311 | HOXA6 | parous/breastfeeding | 5.76E-05 | 0.024 |
| cg27149073 | 5 | 1594330 | SDHAP3 | SDHAP3 | nulliparous/pregnant | 6.73E-05 | 0.056 |
| cg08778598 | 5 | 1594579 | SDHAP3 | SDHAP3 | nulliparous/pregnant | 6.73E-05 | 0.056 |
| cg21167402 | 5 | 1594676 | SDHAP3 | SDHAP3 | nulliparous/pregnant | 6.73E-05 | 0.056 |
| cg24960960 | 5 | 1594678 | SDHAP3 | SDHAP3 | nulliparous/pregnant | 6.73E-05 | 0.056 |
| cg21931717 | 5 | 1594715 | SDHAP3 | SDHAP3 | nulliparous/pregnant | 6.73E-05 | 0.056 |
| cg08422420 | 5 | 1594733 | SDHAP3 | SDHAP3 | nulliparous/pregnant | 6.73E-05 | 0.056 |
| cg06721546 | 5 | 1594808 | SDHAP3 | SDHAP3 | nulliparous/pregnant | 6.73E-05 | 0.056 |
| cg17597639 | 5 | 1594843 | SDHAP3 | SDHAP3 | nulliparous/pregnant | 6.73E-05 | 0.056 |
| cg02248486 | 7 | 27183196 | HOXA5 | HOXA5 | nulliparous/breastfeeding | 1.44E-04 | 0.06 |
| cg25866143 | 7 | 27183262 | HOXA5 | HOXA5;HOXA5 | nulliparous/breastfeeding | 1.44E-04 | 0.06 |
| cg09549073 | 7 | 27183274 | HOXA5 | HOXA5;HOXA5 | nulliparous/breastfeeding | 1.44E-04 | 0.06 |
| cg01370449 | 7 | 27183369 | HOXA5 | HOXA5 | nulliparous/breastfeeding | 1.44E-04 | 0.06 |
| cg04863892 | 7 | 27183375 | HOXA5 | HOXA5 | nulliparous/breastfeeding | 1.44E-04 | 0.06 |
| cg19759481 | 7 | 27183401 | HOXA5 | HOXA5 | nulliparous/breastfeeding | 1.44E-04 | 0.06 |
| cg12128839 | 7 | 27183436 | HOXA5 | HOXA5 | nulliparous/breastfeeding | 1.44E-04 | 0.06 |
| cg02916332 | 7 | 27183591 | HOXA5 | HOXA5 | nulliparous/breastfeeding | 1.44E-04 | 0.06 |
| cg17569124 | 7 | 27183643 | HOXA5 | HOXA5 | nulliparous/breastfeeding | 1.44E-04 | 0.06 |
| cg02005600 | 7 | 27183686 | HOXA5 | HOXA5 | nulliparous/breastfeeding | 1.44E-04 | 0.06 |
| cg25307665 | 7 | 27183694 | HOXA5 | HOXA5 | nulliparous/breastfeeding | 1.44E-04 | 0.06 |
| cg14014955 | 7 | 27183701 | HOXA5 | HOXA5 | nulliparous/breastfeeding | 1.44E-04 | 0.06 |
| cg02646423 | 7 | 27183794 | HOXA5 | HOXA5 | nulliparous/breastfeeding | 1.44E-04 | 0.06 |
| cg20517050 | 7 | 27183806 | HOXA5 | HOXA5 | nulliparous/breastfeeding | 1.44E-04 | 0.06 |
| cg23204968 | 7 | 27183816 | HOXA5 | HOXA5 | nulliparous/breastfeeding | 1.44E-04 | 0.06 |
| cg05835726 | 7 | 27183861 | HOXA5 | HOXA5 | nulliparous/breastfeeding | 1.44E-04 | 0.06 |
| cg14058329 | 7 | 27183946 | HOXA5 | HOXA5 | nulliparous/breastfeeding | 1.44E-04 | 0.06 |
| cg03207666 | 7 | 27183950 | HOXA5 | HOXA5 | nulliparous/breastfeeding | 1.44E-04 | 0.06 |
| cg13512268 | 7 | 27183973 | HOXA5 | HOXA5 | nulliparous/breastfeeding | 1.44E-04 | 0.06 |
| cg23454797 | 7 | 27183990 | HOXA5 | HOXA5 | nulliparous/breastfeeding | 1.44E-04 | 0.06 |
| cg12015737 | 7 | 27184030 | HOXA5 | HOXA5 | nulliparous/breastfeeding | 1.44E-04 | 0.06 |
| cg08070327 | 7 | 27184059 | HOXA5 | HOXA5 | nulliparous/breastfeeding | 1.44E-04 | 0.06 |
| cg25506432 | 7 | 27184065 | HOXA5 | HOXA5 | nulliparous/breastfeeding | 1.44E-04 | 0.06 |
| cg14658493 | 7 | 27184077 | HOXA5 | HOXA5 | nulliparous/breastfeeding | 1.44E-04 | 0.06 |
| cg19196335 | 7 | 27184094 | HOXA5 | HOXA5 | nulliparous/breastfeeding | 1.44E-04 | 0.06 |
| cg09880291 | 7 | 27184109 | HOXA5 | HOXA5 | nulliparous/breastfeeding | 1.44E-04 | 0.06 |
| cg24389585 | 7 | 27184125 | HOXA5 | HOXA5 | nulliparous/breastfeeding | 1.44E-04 | 0.06 |
| cg16997642 | 7 | 27184159 | HOXA5 | HOXA5 | nulliparous/breastfeeding | 1.44E-04 | 0.06 |
| cg20817131 | 7 | 27184167 | HOXA5 | HOXA5 | nulliparous/breastfeeding | 1.44E-04 | 0.06 |
| cg14013695 | 7 | 27184176 | HOXA5 | HOXA5 | nulliparous/breastfeeding | 1.44E-04 | 0.06 |
| cg25390165 | 7 | 27184188 | HOXA5 | HOXA5 | nulliparous/breastfeeding | 1.44E-04 | 0.06 |
| cg01323381 | 7 | 27184264 | HOXA5 | HOXA5 | nulliparous/breastfeeding | 1.44E-04 | 0.06 |
| cg19643053 | 7 | 27184271 | HOXA5 | HOXA5 | nulliparous/breastfeeding | 1.44E-04 | 0.06 |
| cg05774699 | 7 | 27184316 | HOXA5 | HOXA5 | nulliparous/breastfeeding | 1.44E-04 | 0.06 |
| cg26023912 | 7 | 27184369 | HOXA5 | HOXA5 | nulliparous/breastfeeding | 1.44E-04 | 0.06 |
| cg14882265 | 7 | 27184375 | HOXA5 | HOXA5 | nulliparous/breastfeeding | 1.44E-04 | 0.06 |
| cg17432857 | 7 | 27184438 | HOXA5 | HOXA5 | nulliparous/breastfeeding | 1.44E-04 | 0.06 |
| cg00969405 | 7 | 27184441 | HOXA5 | HOXA5 | nulliparous/breastfeeding | 1.44E-04 | 0.06 |
| cg07049592 | 7 | 27184450 | HOXA5 | HOXA5 | nulliparous/breastfeeding | 1.44E-04 | 0.06 |
| cg02106682 | 7 | 27184461 | HOXA5 | HOXA5 | nulliparous/breastfeeding | 1.44E-04 | 0.06 |
| cg03368099 | 7 | 27184521 | HOXA5 | HOXA5 | nulliparous/breastfeeding | 1.44E-04 | 0.06 |
| cg01748892 | 7 | 27184667 | HOXA5 | HOXA5 | nulliparous/breastfeeding | 1.44E-04 | 0.06 |
| cg13694927 | 7 | 27184712 | HOXA5 | HOXA5 | nulliparous/breastfeeding | 1.44E-04 | 0.06 |
| cg03744763 | 7 | 27184737 | HOXA5 | HOXA5 | nulliparous/breastfeeding | 1.44E-04 | 0.06 |
| cg27151303 | 7 | 27184821 | HOXA5 | NA | nulliparous/breastfeeding | 1.44E-04 | 0.06 |
| cg05579037 | 7 | 27184853 | HOXA5 | NA | nulliparous/breastfeeding | 1.44E-04 | 0.06 |
| cg21641458 | 7 | 27185136 | LOC100133311 | HOXA6 | nulliparous/breastfeeding | 1.44E-04 | 0.06 |
| cg09343092 | 7 | 27185282 | LOC100133311 | HOXA6 | nulliparous/breastfeeding | 1.44E-04 | 0.06 |
| cg18931036 | 7 | 27185393 | LOC100133311 | HOXA6 | nulliparous/breastfeeding | 1.44E-04 | 0.06 |
| cg03293507 | 6 | 30038975 | PPP1R11 | RNF39;RNF39 | parous/pregnant | 4.31E-04 | 0.18 |
| cg16582419 | 6 | 30038998 | PPP1R11 | RNF39;RNF39 | parous/pregnant | 4.31E-04 | 0.18 |
| cg23793213 | 6 | 30039006 | PPP1R11 | RNF39;RNF39 | parous/pregnant | 4.31E-04 | 0.18 |
| cg12736877 | 6 | 30039010 | PPP1R11 | RNF39;RNF39 | parous/pregnant | 4.31E-04 | 0.18 |
| cg24637044 | 6 | 30039013 | PPP1R11 | RNF39;RNF39 | parous/pregnant | 4.31E-04 | 0.18 |
| cg01286685 | 6 | 30039025 | PPP1R11 | RNF39;RNF39 | parous/pregnant | 4.31E-04 | 0.18 |
| cg05563515 | 6 | 30039027 | PPP1R11 | RNF39;RNF39 | parous/pregnant | 4.31E-04 | 0.18 |
| cg08491487 | 6 | 30039130 | PPP1R11 | RNF39;RNF39 | parous/pregnant | 4.31E-04 | 0.18 |
| cg23712018 | 6 | 30039132 | PPP1R11 | RNF39;RNF39 | parous/pregnant | 4.31E-04 | 0.18 |
| cg00947782 | 6 | 30039142 | PPP1R11 | RNF39;RNF39 | parous/pregnant | 4.31E-04 | 0.18 |
| cg18930910 | 6 | 30039151 | PPP1R11 | RNF39;RNF39 | parous/pregnant | 4.31E-04 | 0.18 |
| cg03343571 | 6 | 30039175 | PPP1R11 | RNF39;RNF39 | parous/pregnant | 4.31E-04 | 0.18 |
| cg13185413 | 6 | 30039202 | PPP1R11 | RNF39;RNF39 | parous/pregnant | 4.31E-04 | 0.18 |
| cg06249604 | 6 | 30039206 | PPP1R11 | RNF39;RNF39 | parous/pregnant | 4.31E-04 | 0.18 |
| cg20119745 | 6 | 30039239 | PPP1R11 | RNF39;RNF39 | parous/pregnant | 4.31E-04 | 0.18 |
| cg20249327 | 6 | 30039374 | RNF39 | RNF39;RNF39 | parous/pregnant | 4.31E-04 | 0.18 |
| cg15877520 | 6 | 30039376 | RNF39 | RNF39;RNF39 | parous/pregnant | 4.31E-04 | 0.18 |
| cg13918754 | 6 | 30039380 | RNF39 | RNF39;RNF39 | parous/pregnant | 4.31E-04 | 0.18 |
| cg09279736 | 6 | 30039403 | RNF39 | RNF39;RNF39 | parous/pregnant | 4.31E-04 | 0.18 |
| cg07382347 | 6 | 30039408 | RNF39 | RNF39;RNF39 | parous/pregnant | 4.31E-04 | 0.18 |
| cg13401893 | 6 | 30039432 | RNF39 | RNF39;RNF39 | parous/pregnant | 4.31E-04 | 0.18 |
| cg12633154 | 6 | 30039435 | RNF39 | RNF39;RNF39 | parous/pregnant | 4.31E-04 | 0.18 |
| cg10568066 | 6 | 30039442 | RNF39 | RNF39;RNF39 | parous/pregnant | 4.31E-04 | 0.18 |
| cg16078649 | 6 | 30039466 | RNF39 | RNF39;RNF39 | parous/pregnant | 4.31E-04 | 0.18 |
| cg10930308 | 6 | 30039476 | RNF39 | RNF39;RNF39 | parous/pregnant | 4.31E-04 | 0.18 |
| cg02188185 | 6 | 30039524 | RNF39 | RNF39;RNF39 | parous/pregnant | 4.31E-04 | 0.18 |
| cg07179033 | 6 | 30039548 | RNF39 | RNF39;RNF39 | parous/pregnant | 4.31E-04 | 0.18 |
| cg26730543 | 6 | 30039600 | RNF39 | RNF39;RNF39 | parous/pregnant | 4.31E-04 | 0.18 |
| cg24099074 | 11 | 34460172 | CAT | CAT | nulliparous/parous | 0.00158516 | 0.532 |
| cg22159421 | 11 | 34460182 | CAT | CAT | nulliparous/parous | 0.00158516 | 0.532 |
| cg20731136 | 11 | 34460298 | CAT | CAT | nulliparous/parous | 0.00158516 | 0.532 |
| cg06027906 | 11 | 34460318 | CAT | CAT | nulliparous/parous | 0.00158516 | 0.532 |
| cg07768201 | 11 | 34460336 | CAT | CAT | nulliparous/parous | 0.00158516 | 0.532 |
| cg03720043 | 11 | 34460351 | CAT | CAT | nulliparous/parous | 0.00158516 | 0.532 |
| cg02109652 | 11 | 34460386 | CAT | CAT | nulliparous/parous | 0.00158516 | 0.532 |
| cg06908474 | 11 | 34460516 | CAT | CAT;CAT | nulliparous/parous | 0.00158516 | 0.532 |
| cg01847719 | 11 | 34460557 | CAT | CAT;CAT | nulliparous/parous | 0.00158516 | 0.532 |
| cg14316565 | 11 | 34460789 | CAT | CAT | nulliparous/parous | 0.00158516 | 0.532 |
| cg03728580 | 11 | 34460856 | CAT | CAT | nulliparous/parous | 0.00158516 | 0.532 |

**Supplementary References**

Benjamini, Y., & Hochberg, Y. (1995). Controlling the false discovery rate: A practical and powerful approach to multiple testing. *Journal of the Royal Statistical Society. Series B (Methodological)*, 289–300.

Bourgon, R., Gentleman, R., & Huber, W. (2010). Independent filtering increases detection power for high-throughput experiments. *Proceedings of the National Academy of Sciences*, *107*(21), 9546–9551. https://doi.org/10.1073/pnas.0914005107

Du, P., Zhang, X., Huang, C.-C., Jafari, N., Kibbe, W. A., Hou, L., & Lin, S. M. (2010). Comparison of Beta-value and M-value methods for quantifying methylation levels by microarray analysis. *BMC Bioinformatics*, *11*, 587. https://doi.org/10.1186/1471-2105-11-587

Fishilevich, S., Nudel, R., Rappaport, N., Hadar, R., Plaschkes, I., Iny Stein, T., … Cohen, D. (2017). GeneHancer: Genome-wide integration of enhancers and target genes in GeneCards. *Database: The Journal of Biological Databases and Curation*, *2017*. https://doi.org/10.1093/database/bax028

Gillis, J., Mistry, M., & Pavlidis, P. (2010). Gene function analysis in complex data sets using ErmineJ. *Nature Protocols*, *5*(6), 1148–1159. https://doi.org/10.1038/nprot.2010.78

Gillis, J., & Pavlidis, P. (2011). The Impact of Multifunctional Genes on “Guilt by Association” Analysis. *PLOS ONE*, *6*(2), e17258. https://doi.org/10.1371/journal.pone.0017258

Hansen, K. (2015). IlluminaHumanMethylation450kanno. Ilmn12. Hg19: Annotation for illumina’s 450k methylation arrays. *R Package, Version 0.2*, *1*.

Houseman, E. A., Accomando, W. P., Koestler, D. C., Christensen, B. C., Marsit, C. J., Nelson, H. H., … Kelsey, K. T. (2012). DNA methylation arrays as surrogate measures of cell mixture distribution. *BMC Bioinformatics*, *13*(1), 86. https://doi.org/10.1186/1471-2105-13-86

Jaffe, A. E., Murakami, P., Lee, H., Leek, J. T., Fallin, M. D., Feinberg, A. P., & Irizarry, R. A. (2012). Bump hunting to identify differentially methylated regions in epigenetic epidemiology studies. *International Journal of Epidemiology*, *41*(1), 200–209. https://doi.org/10.1093/ije/dyr238

Jones, M. J., Islam, S. A., Edgar, R. D., & Kobor, M. S. (2015). Adjusting for Cell Type Composition in DNA Methylation Data Using a Regression-Based Approach. In P. Haggarty & K. Harrison (Eds.), *Population Epigenetics* (Vol. 1589, pp. 99–106). New York, NY: Springer New York. https://doi.org/10.1007/7651_2015_262

Leek, J. T., Johnson, W. E., Parker, H. S., Jaffe, A. E., & Storey, J. D. (2012). The sva package for removing batch effects and other unwanted variation in high-throughput experiments. *Bioinformatics*, *28*(6), 882–883.

Maksimovic, J., Gordon, L., & Oshlack, A. (2012). SWAN: Subset-quantile Within Array Normalization for Illumina Infinium HumanMethylation450 BeadChips. *Genome Biology*, *13*(6), R44. https://doi.org/10.1186/gb-2012-13-6-r44

McDade, T. W., Ryan, C. P., Jones, M. J., Hoke, M. K., Borja, J., Miller, G. E., … Kobor, M. S. (2019). Genome-wide analysis of DNA methylation in relation to socioeconomic status during development and early adulthood. *American Journal of Physical Anthropology*, *169*(1), 3–11. https://doi.org/10.1002/ajpa.23800

Merico, D., Isserlin, R., Stueker, O., Emili, A., & Bader, G. D. (2010). Enrichment Map: A Network-Based Method for Gene-Set Enrichment Visualization and Interpretation. *PLOS ONE*, *5*(11), e13984. https://doi.org/10.1371/journal.pone.0013984

Mill, J., & Heijmans, B. T. (2013). From promises to practical strategies in epigenetic epidemiology. *Nature Reviews Genetics*, *14*(8), 585–594. https://doi.org/10.1038/nrg3405

Morris, T. J., Butcher, L. M., Feber, A., Teschendorff, A. E., Chakravarthy, A. R., Wojdacz, T. K., & Beck, S. (2014). ChAMP: 450k Chip Analysis Methylation Pipeline. *Bioinformatics*, *30*(3), 428–430. https://doi.org/10.1093/bioinformatics/btt684

Price, E. M., Cotton, A. M., Lam, L. L., Farré, P., Emberly, E., Brown, C. J., … Kobor, M. S. (2013). Additional annotation enhances potential for biologically-relevant analysis of the Illumina Infinium HumanMethylation450 BeadChip array. *Epigenetics & Chromatin*, *6*(1), 1.

Rakyan, V. K., Down, T. A., Balding, D. J., & Beck, S. (2011). Epigenome-wide association studies for common human diseases. *Nature Reviews Genetics*, *12*(8), 529–541.

Rappaport, N., Twik, M., Plaschkes, I., Nudel, R., Iny Stein, T., Levitt, J., … Lancet, D. (2017). MalaCards: An amalgamated human disease compendium with diverse clinical and genetic annotation and structured search. *Nucleic Acids Research*, *45*(D1), D877–D887. https://doi.org/10.1093/nar/gkw1012

Ritchie, M. E., Phipson, B., Wu, D., Hu, Y., Law, C. W., Shi, W., & Smyth, G. K. (2015). Limma powers differential expression analyses for RNA-sequencing and microarray studies. *Nucleic Acids Research*, *43*(7), e47–e47. https://doi.org/10.1093/nar/gkv007

Shannon, P. (2003). Cytoscape: A Software Environment for Integrated Models of Biomolecular Interaction Networks. *Genome Research*, *13*(11), 2498–2504. https://doi.org/10.1101/gr.1239303

Stelzer, G., Rosen, N., Plaschkes, I., Zimmerman, S., Twik, M., Fishilevich, S., … Lancet, D. (2016). The GeneCards Suite: From Gene Data Mining to Disease Genome Sequence Analyses. *Current Protocols in Bioinformatics*, *54*(1), 1.30.1-1.30.33. https://doi.org/10.1002/cpbi.5

Uhlén, M., Fagerberg, L., Hallström, B. M., Lindskog, C., Oksvold, P., Mardinoglu, A., … Pontén, F. (2015). Tissue-based map of the human proteome. *Science*, *347*(6220). https://doi.org/10.1126/science.1260419

Zeilinger, S., Kühnel, B., Klopp, N., Baurecht, H., Kleinschmidt, A., Gieger, C., … Illig, T. (2013). Tobacco Smoking Leads to Extensive Genome-Wide Changes in DNA Methylation. *PLOS ONE*, *8*(5), e63812. https://doi.org/10.1371/journal.pone.0063812
